# Peripartum Cardiomyopathy

**DOI:** 10.21980/J8ZS9M

**Published:** 2023-04-30

**Authors:** Victoria L Morris, Carolina Mendoza, Gowri S Stevens, Jessica L Wilson, Adeola A Kosoko

**Affiliations:** * University of Texas Health Sciences Center at Houston, Department of Emergency Medicine, Houston, TX

## Abstract

**Audience:**

This simulation is appropriate for emergency medicine (EM) residents of all levels.

**Introduction:**

Peripartum cardiomyopathy (PPCM) is a rare, idiopathic condition that occurs in the mother around the time of childbirth. Heart failure with reduced ejection fraction and/or reduced systolic function diagnosed in patients during the last month of pregnancy or up to five months following delivery defines PCCM.^1^ Another broader definition from the European Society of Cardiology defines PPCM as heart failure that occurs “towards the end of pregnancy or in the months following delivery, where no other cause of heart failure is found.”^2^ Though PPCM occurs worldwide, most data is extracted from the United States (incidence 1:900 to 1:4000 live births), Nigeria, Haiti, and South Africa.^3^,^4^

Risk factors for PPCM include pre-eclampsia, multiparity, and advanced maternal age. Unfortunately, the complete pathophysiology of PPCM remains unclear. However, it is important for emergency physicians to be aware of this rare diagnosis because though 50–80% of women with PPCM may eventually recover normal left ventricle systolic function,^5^ positive outcomes depend on timely recognition of PPCM as a disease and the appropriate management of heart failure. Symptomatic PPCM is an emergent condition that requires an attentive and knowledgeable emergency medicine physician for rapid recognition and treatment. A simulation of this rare condition can give residents the experience of identifying and managing this disease that they might not otherwise see personally during their training.

**Educational Objectives:**

By the end of this simulation session, learners will be able to: 1) initiate a workup of a pregnant patient who presents with syncope, 2) accurately diagnose peripartum cardiomyopathy, 3) demonstrate care of a gravid patient in respiratory distress due to peripartum cardiomyopathy, 4) appropriately manage cardiogenic shock due to peripartum cardiomyopathy.

**Educational Methods:**

This simulation was conducted as a high-fidelity medical simulation case followed by a debriefing. It could potentially be adapted for use as a low-fidelity case or an oral boards exam case.

**Research Methods:**

The educational content and clinical applicability of this simulation was evaluated by oral and written feedback from participant groups at a large three-year emergency medicine residency training program. Each participant completed the case and the facilitated debriefing afterwards. Case facilitators also provided their personal observations on the implementation of the simulation.

**Results:**

The participants gave the simulation positive feedback (n=18). Seventeen EM residents and one pediatric emergency medicine (PEM) fellow participated in the feedback survey. Learners overall agreed (18.75%) or strongly agreed (81.25%) that participating in this simulation would improve their performance in a live clinical setting.

**Discussion:**

Peripartum cardiomyopathy is a low frequency, high acuity illness that requires a synthesis of the learner’s knowledge of complex physiology, navigation of logistical and systems-based challenges, and advanced communication and leadership skills to ensure the best possible patient outcome. All EM physicians will be expected to expertly manage this illness after completion of an EM training program, yet not every EM resident will encounter this type of patient during training. Supplementing the EM resident’s standard training with this simulation experience provides a psychologically and educationally safe space to learn and possibly make mistakes without causing patient harm. Practically all residents were able to correctly diagnose the patient with a cardiomyopathy even if they were not familiar with the diagnosis of “peripartum cardiomyopathy.” The residents particularly enjoyed the case to explore concepts of benefits and risks of medical therapeutics (ie, positive pressure ventilation, vasopressors/inotropes) and safe practice for the gravid patient. This case and the associated high yield debriefing session were effective teaching tools for emergency medicine residents about PPCM.

**Topics:**

Medical simulation, peripartum cardiomyopathy, pregnancy, respiratory failure, cardiogenic shock, emergent cesarian section.

## USER GUIDE

**Table t4-jetem-8-2-s1:** 

List of Resources:	
Abstract	1
User Guide	3
Instructor Materials	6
Operator Materials	20
Debriefing and Evaluation Pearls	25
Simulation Assessment	30

[Table t4-jetem-8-2-s1] **Learner Audience:**

Interns, Junior Residents, Senior Residents

**Time Required for Implementation:**

Instructor Preparation: 20 minutes Time for case: 15–20 minutes Time for debriefing: 15–30 minutes

**Recommended Number of Learners per Instructor:**

3–5 learners per instructor, 1 instructor per case

**Topics:**

Acute exacerbation COPD, intubation, positive pressure ventilation, ventilator alarms, chest tube thoracostomy.

**Objectives:**

By the end of this simulation session, learners will be able to:

1. Initiate a workup of a pregnant patient who presents with syncope
2. Accurately diagnose peripartum cardiomyopathy
3. Demonstrate care of a gravid patient in respiratory distress due to peripartum cardiomyopathy
4. Appropriately manage cardiogenic shock due to peripartum cardiomyopathy

### Linked objectives and methods

Peripartum cardiomyopathy is a rare condition that has the potential for significant morbidity and mortality for an obstetric patient. Clinical presentation is often under-recognized because symptoms are often attributed to signs and symptoms of normal pregnancy. However, it is important for the emergency physician to maintain a high level of suspicion for this rare disease to be able to diagnose and appropriately treat PPCM. This case simulates a pregnant patient presenting via emergency medical services (EMS) in respiratory distress with a history and exam concerning for PPCM. Learners must begin assessing and treating a gravid patient presenting with syncope in respiratory distress (Objective 1 and 3). They need to perform a diagnostic work up for the presenting complaint, form a differential diagnosis, initiate fetal monitoring, and consult subspecialty services. Learners must recognize the signs and symptoms of respiratory failure from pulmonary edema and initiate diagnostic studies to rule in/rule out PPCM (Objective 2 and 3). They should recognize the signs of cardiogenic shock and appropriately treat the patient’s cardiogenic shock (Objectives 3 and 4). The simulation is complete when the patient is admitted to either the intensive care unit (ICU) or is transported to the operating room (OR) for emergent delivery with obstetric consultants.

### Recommended pre-reading for instructor

Rometti M, Patti L. Peripartum cardiomyopathy – ED Presentation, evaluation, and management. emDOCs.net-Emergency Medicine Education. Published July 4, 2019. Accessed August 28, 2021. At: https://www.emdocs.net/peripartum-cardiomyopathy-edpresentation-evaluation-and-management/

### Results and tips for successful implementation

This case simulation was designed for residents to diagnose, treat, and appropriately disposition a patient with peripartum cardiomyopathy, cardiogenic shock, and respiratory failure in the emergency department. This scenario was designed to be performed using a high-fidelity simulation setup and manikin. The case allows learners to work through a broad differential diagnosis for syncope as well as hypotension in late pregnancy. Management of shock and respiratory distress are explored in this case.

This case was conducted in groups of three to five learners for a total of 17 EM residents and one pediatric EM fellow. We used a Laerdal SimMan 3G and applied a commercially available, silicone artificial pregnancy belly and long-haired wig for moulage. Of note, our pregnancy belly moulage had previously been modified from another simulation case for which it signified an abdomen with ascites and caput medusa. We used this moulage/prosthetic to mimic a gravid abdomen by placing it under the manikin gown. A facilitator voiced the patient, consultants, and facilitated data sharing. A confederate acted as the patient’s mother and kept observational field notes of learner performance for debriefing.

A convenience sample of 16 participants completed a feedback survey including EM residents (n=15) and a PEM fellow (n=1) (Table 1). The survey consisted of nine questions; five questions that each used a 5-point Likert scale (Table 2) and two questions requested open-ended responses. Feedback was overall positive regarding the simulation case and the debriefing session. All learners felt that participation in this medical simulation would contribute to improved performance in the live clinical setting (“strongly agree” or “agree”). Sample responses to the open-ended questions are provided in Table 3.

On facilitator observation and per participant feedback, the use of a simulation ascitic abdomen as moulage for a gravid abdomen was distracting for participants, who confused what was meant to be a gravid abdomen with an ascitic abdomen on their physical exam. Future iterations of this case would benefit from a more clearly labelled moulage or higher fidelity moulage.

We also observed that residents inconsistently intervened upon the patient’s tachypnea. It was unclear to the facilitators if learners had real time situational awareness of this vital sign abnormality. During debrief, almost all learners stated they were aware of the elevated respiratory rate. This demonstrates one limitation of this case: the manikin had an elevated respiratory rate but cannot imitate retractions, use of accessory muscles or “tripoding.” It is possible that this limitation obscured the need for further respiratory support to a learner. When the facilitators notice that the participants are not intervening, the confederate playing the mother or the nurse can comment on her daughter’s respiratory efforts and distress, although, a more well-versed EM physician may be more inclined to intervene on the concerning vital sign given the patient’s clinical picture.

Residents rarely placed early supplemental oxygen for initial oxygen saturation (O2sat) of 94% though the patient was tachypneic and they indeed identified the patient was in shock. The facilitator, on most occasions, had to worsen the O2sat and respiratory rate to elicit a respiratory intervention. When asked about early respiratory intervention during debriefing, one resident explained “It’s not *that* bad and we see that type of O2sat all the time, especially with patients who have [SARS-CoV-2]. Maybe I’ve become numb.”

The patient, the mother, and EMS are all potential sources of history for the patient. Only two of the six groups spoke with EMS to obtain further history. A confederate (playing the patient’s mother) was present in the room to provide the history of the syncope. Only one group elicited history from the mother, who witnessed the syncopal event in the scenario. A potential barrier to questioning the EMS may have been that there was not a physical presence of a confederate prehospital worker; rather, the facilitator provided the prehospital history if asked. Future iterations of this simulation should prompt interaction with the confederates by a simple removable costume or clothing label (eg, a hazard vest or nametag). During the physical exam and diagnostic portion of the simulation, all groups asked for a stimulus of bedside ultrasound images of the heart and lungs. Each group quickly identified decreased ejection fraction and cardiac dysfunction. Of the six groups, only one group placed the patient on bi-level positive airway pressure ventilation (BPAP). The more common respiratory intervention was supplementary oxygen. Some groups discussed the use of BPAP but decided against its use due to concern that increasing intrathoracic pressures would decrease the patient’s already low blood pressure. Specifically, more senior residents tended to overestimate the risk of BPAP potentially causing hypotension and underestimated the benefit of supporting the patient’s respiratory distress. All groups gave the patient intravenous vasopressors for circulatory support. Five of the six groups chose norepinephrine as their initial vasopressor.

All groups consulted obstetrics regarding the care of this patient, but only three groups asked for continuous cardiotocography for fetal monitoring.

All groups appropriately admitted the patient to the intensive care unit (ICU).

In future iterations, we plan to adjust the post-simulation survey to ask more specific questions to assess the resident’s depth of knowledge in addition to facilitator feedback to have a better understanding of this simulation’s impact on the learners.

## Supplementary Information





## Figures and Tables

**Table t5-jetem-8-2-s1:** 

#1	Point of Care Glucose
#2	Complete Blood Count (CBC)
#3	Basic Metabolic Profile (BMP)
#4	Liver Function Panel (LFT)
#5	Venous Blood Gas (VBG) and Lactate
#6	Coagulopathy Panel
#7	Fibrinogen and D-Dimer
#8	Lactate dehydrogenase (LDH), Uric acid
#9	Troponin-I
#10	Brain Natriuretic Peptide (BNP)
#11	Urine Pregnancy
#12	Urinalysis (UA)
#13	Urine Drug Screen
#14	Electrocardiogram (ECG)
#15	Chest X-ray
#16	Point of Care Echocardiogram
#17	Computed Tomography (CT) of Head
#18	Cardiotocography tracing

**Table t6-jetem-8-2-s1:** 

**Vitals:** Heart Rate (HR) 130	Blood Pressure (BP) 89/60	Respiratory Rate (RR) 26
Temperature (T) 99.7°F	Oxygen Saturation (O_2_Sat) 93% on room air	

**Table t7-jetem-8-2-s1:** 

*Point of case glucose*	93mg/dL
*Complete blood count (CBC)*	
White blood count (WBC)	16.0 ×1000/mm^3^
Hemoglobin (Hgb)	12.0 g/dL
Hematocrit (HCT)	36.0%
Platelet (Plt)	280 ×1000/mm^3^
Differential	pending
*Basic metabolic panel (BMP)*	
Sodium	140 mEq/L
Chloride	109 mEq/L
Potassium	3.8 mEq/L
Bicarbonate (HCO_3_)	25 mEq/L
Blood Urea Nitrogen (BUN)	45 mg/dL
Creatine (Cr)	1.9 mg/dL
Glucose	90 mg/dL
Calcium	9.1 mg/dL
*Liver function panel*	
Total bilirubin	0.5 mg/dL
Alkaline phosphatase	60 units/L
Aspartate aminotransferase	105 units/L
Alanine aminotransferase	95 units/L
Albumin	3.6 units/L
*Venous blood gas (VBG)*	
pH	7.35
pCO2	25 mmHg
pO2	50 mmHg
HCO3	15 mEq/L
Lactic Acid	3.9 mEq/L
*Coagulopathy Panel*	
International Normalized Ration (INR)	1.14
Prothrombin Time (PT)	12.3 seconds
Activated Partial Thromboplastin Time (aPTT)	23.7 seconds
*Fibrinogen*	400 mg/mL
*D-Dimer*	0.98 mg/L
*Lactate dehydrogenase (LDH)*	270 units/L
*Uric Acid*	5.0 mg/L
*Troponin-I*	0.50 ng/mL
*Brain Natriuretic Peptide (BNP)*	*positive*
*Urine pregnancy*	*positive*
*Urinalysis (UA)*	
Color	yellow
Appearance	clear
Specific gravity	1.010
pH	7.0
Glucose	negative
Bilirubin	negative
Ketones	0
Protein	1+
Leukocyte esterase	1+
Nitrites	negative
White blood cells (WBC)	1 cells/high powered field (HPF)
Red blood cells (RBC)	0 cells /HPF
Squamous epithelial cells	0–5 cells/HPF
*Urine Toxicology Screen:*	
Alcohol	negative
Amphetamines	negative
Barbiturates	negative
Benzodiazepines	negative
Cocaine	negative
Marijuana	negative
Nicotine	negative
Opiates	negative
Phencyclidine (PCP)	negative

**Figure f1-jetem-8-2-s1:**
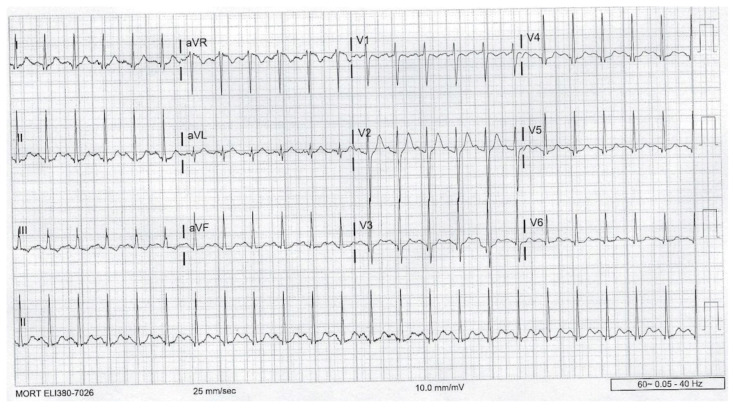
[Fig f1-jetem-8-2-s1]Electrocardiogram (ECG) Image Source: Author’s Own Image

**Figure f2-jetem-8-2-s1:**
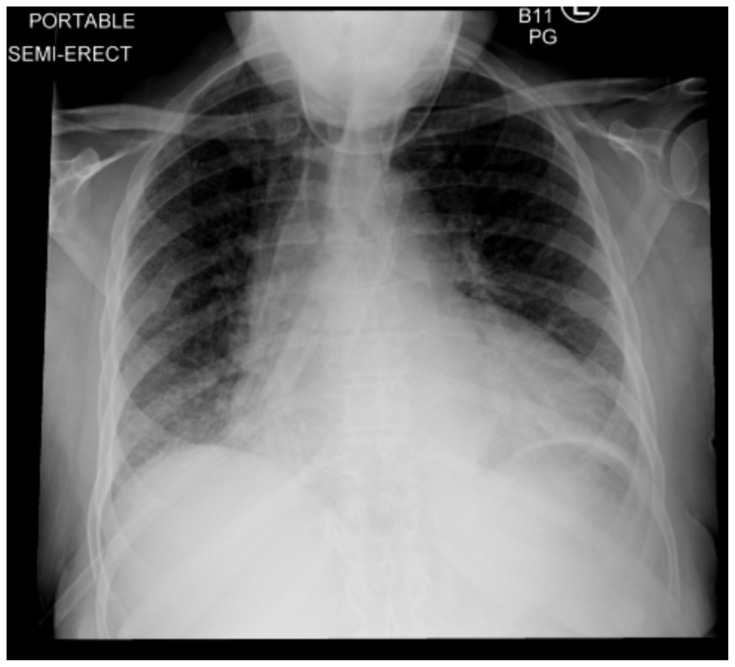
[Fig f2-jetem-8-2-s1] *Chest radiograph* Image Source: Author’s Own Image

**Figure f3-jetem-8-2-s1:**
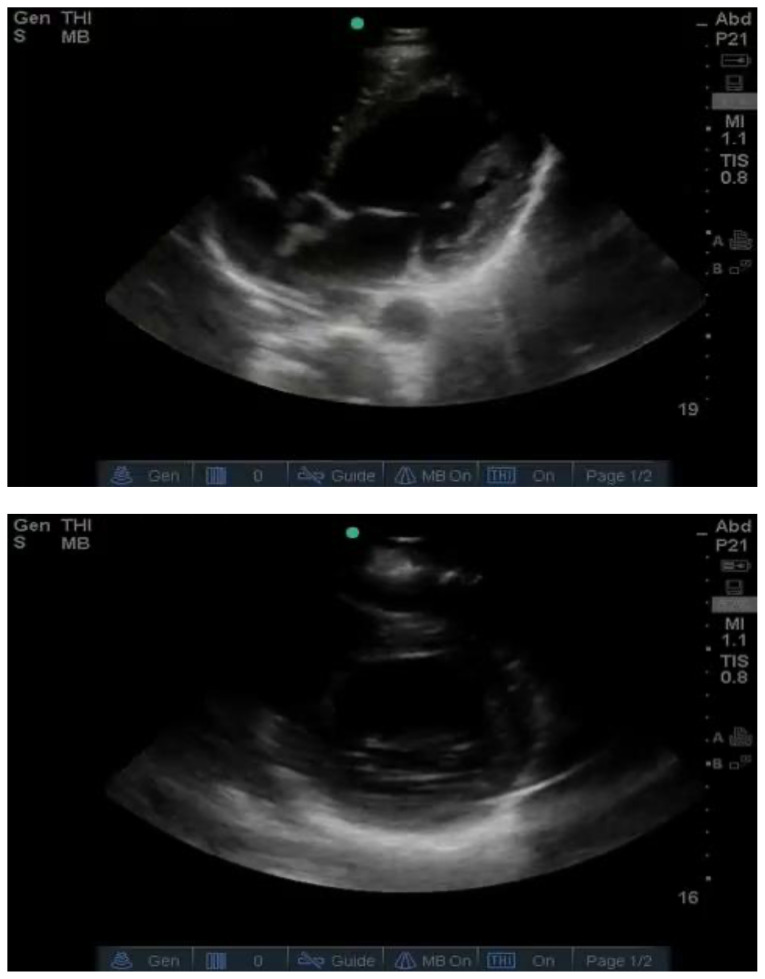
[Fig f3-jetem-8-2-s1]Point of Care Echocardiogram Image Source: Author’s Own Image

**Figure f4-jetem-8-2-s1:**
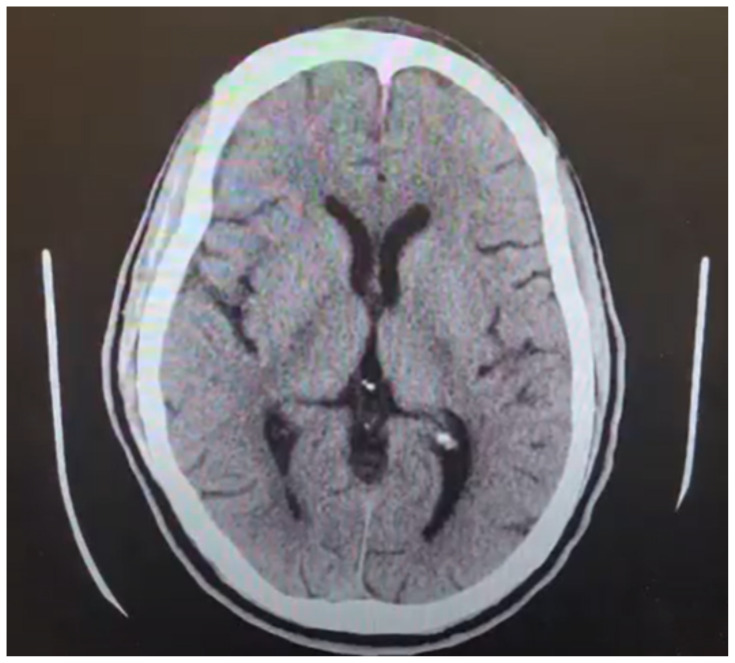
[Fig f4-jetem-8-2-s1]Computed Tomography (CT) of Head without Contrast-Axial Image Source: Author’s Own Image

**Figure f5-jetem-8-2-s1:**
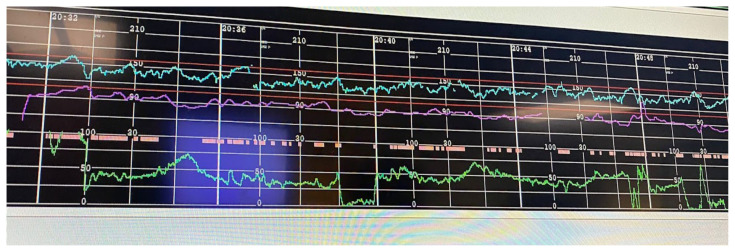
Cardiotocography [Fig f5-jetem-8-2-s1]Image Source: Author’s Own Image[Fig f5-jetem-8-2-s1]

**Table t8-jetem-8-2-s1:** SIMULATION EVENTS TABLE:

Minute (state)	Participant action/ trigger	Patient status (simulator response) & operator prompts	Monitor display (vital signs)
0:00 (Baseline)	Introductions Attach the patient to cardiac monitor and pulse oximeter Obtain vital signs EMS report Perform primary survey	Patient was brought in by EMS with a chief complaint of syncope Patient appears in respiratory distress and is hypoxic *Patient is in moderate respiratory distress, so answers should be short. The patient is unable to state complete sentences, pausing to take breaths*.	T 99.7° F HR 130 BP 89/48 RR 26 O2 93%
If learners ask for **EMS report**: “This is a 36-year-old pregnant woman with no known past medical history presenting with syncopal episode at home. The episode was witnessed by family, who are on the way. Per family, the patient stood up, then passed out. She was unresponsive for about 10 seconds. She did not hit her head. There was no reported seizure-like activity. The vital signs on scene were heart rate 130s, blood pressure 95/65, respiratory rate 24, temperature 99.2° F, saturation was 94% on room air. Point of care glucose was 102 on scene.”			
2:00	Obtain full history and physical exam Obtain IV access Give supplemental O2 Order laboratory and imaging studies	Patient will have a normal neurological exam with no acute neurological deficits. *If supplemental oxygen is not provided in first 3 minutes:* ***Nurse****: “Doc, the oxygen sats look low.”* *When the patient saturations are improved on nasal cannula but the patient is still in respiratory distress:* ***Parent****: “I don’t think she is getting enough air.”* *If no labs or imaging is ordered:* ***Nurse****: “What labs would you like me to draw?”* *If supplemental oxygen is not provided in first 3 minutes:* ***Parent:**** “Why is she breathing so hard? What do those numbers mean?”*	T 99.7° F HR 130 BP 89/48 RR 26 O2: 93% on 95% NC 98% on NRB
4:00	Recognize pregnancy and hypotension; place in left lateral tilt with wedge Administer isotonic fluid bolus for hypotension	If hypotension is addressed and IV fluids and/or positioning is attempted: Blood pressure will mildly improve but the patient will remain tachycardic. If hypotension is addressed and IV fluids and/or positioning is not attempted: Blood pressure will remain low, and the patient will remain tachycardic. *If hypotension and tachycardia are not addressed:* ***Nurse:**** “I think we should do something about that blood pressure.”*	If given fluids/ positioned: T 99.7°F HR 140 BP 91/49 RR 28 O2 95% If not given fluids/ positioned: T 99.7° F HR 128 BP 88/48 RR 26 O2 95%
5:00	Noninvasive positive pressure ventilation (NPPV) or intubation for continued respiratory distress and hypoxia Point of care ultrasound (POCUS) of heart and abdomen Obtain fetal heart rate (FHR) Patient will be pending radiographic studies Laboratory studies will slowly result	The patient is no longer hypoxic while receiving supplementary O2, but she has increased work of breathing which can be addressed by NPPV or mechanical ventilation. POCUS (if obtained) will suggest decreased left ventricular function. POCUS of the abdomen will demonstrate an intrauterine pregnancy with a heart rate (FHR) of 165 beats per minute. FHR could also be obtained by doppler. *If fetal heart tones are not checked:* *Nurse: “Would you like me to get the doppler or ultrasound to check on the baby?”* *If fetal heart tones are not checked:* *Parent: “Is the baby okay?”* *If learners request doppler fetal heart tones:* ***Nurse****: “162 beats/min.”* *If learners do not request a point of care ultrasound:* ***Nurse****: “Both of her legs are edematous; do you want to take a look at her cardiac function?”* *When the patient saturations are improved on nasal cannula, but the patient is still in respiratory distress:* ***Nurse****: “The patient is still not breathing well; any other ideas?”* *If the learners provide another supplemental oxygenation besides NIPPV or intubation: ****Nurse****: “The patient is still working very hard to breathe.”* *If NIPPV/ventilator settings are not stated:* ***Nurse****: “What ventilator settings should be set?”*	If given NPPV or mechanical intubation: T 99.7° F HR 115 BP 88/48 RR 22 O2 96% If not NPPV or mechanical intubation: T 99.7°F HR 138 BP 89/48 RR 30 O2 93%
7:00	Review laboratory findings and imaging	Chest x-ray shows cardiomegaly with pulmonary edema. *If learners request any laboratory studies which are not available as a stimulus:* ***Nurse****: “Yes, I’ll make sure to get that sent to the lab,” or “The lab says that they’re still working on it.”* *If learners request any point of care ultrasound studies which are not available as a stimulus:* ***Nurse****: “The Focused Abdominal Exam in Trauma (FAST exam) is negative.” Or: “The Rapid Exam for Shock or Hypotension (RUSH) is positive for the findings you can see on the ultrasound images presented (cardiac windows in the stimulus materials).”*	
8:00	Following return of radiographic imaging, the participants should identify that this patient likely has PPCM Consult obstetrics	The diagnosis cannot be definitively made until other conditions have been ruled out; however, the data presented should strongly suggest PPCM. *If the leading differential diagnoses are not stated during case:* ***Nurse****: “What do we think is the diagnosis?”* *If differential diagnosis is not stated during case:* ***Parent****: “What is going on, doc?”* Obstetrics should have been notified by this point for potential emergency C-section and at minimum for fetal heart rate monitoring. If learners do not call Obstetrics: **Parent**: “Should I call her OB?” *If learners do not call Obstetrics:* ***Nurse****: “Do you think we should call obstetrics?”*	T 99.7°F HR 120 BP 89/48 RR 30 O2 93%
Obstetrician Script “Hello, this is Dr. Toco.” *Learners request consult and describe case* “Keep supporting her hemodynamics with inotropes/vasopressors and respiratory support. We have sent a nurse to begin continuous fetal monitoring. You can look at the tracing if you’d like. We will plan for a likely emergent C-section. Have you spoken with cardiology?”			
10:00	Initiate vasopressors/inot ropes for cardiogenic shock Diuretic administration Consult cardiology	The patient will be hypotensive, tachycardic, fatigued, and with continued respiratory distress. If the learners do not recognize cardiogenic shock, the patient should become more fatigued. *If learners do not start vasopressors and inotropes for persistent hypotension:* ***Parent****: “I think she is passing out again.”* *If learners do not start vasopressors and inotropes for persistent hypotension:* ***Nurse****: “The patient is becoming less responsive; the blood pressure is continuing to drop. Do you want to start anything?”* It is permissible to begin vasopressors with a peripheral IV line. Some learners will place an arterial line and a central venous line. Discussion should be had with cardiology for potential for invasive testing and the potential for cardiac assist devices. *If learners do not call cardiology:* ***Nurse****: “Do you think we should call cardiology?”* *If learners do not call cardiology:* ***Parent****: “You keep talking about her heart; do I need to call my cardiologist?”*	If given vasopressors: T 99.7° F HR 115 BP 98/69 RR 22 O2 98% If not given vasopressors: T 99.7° F HR 139 BP 68/48 RR 22 O2 94%
Cardiologist Script “Hello, this is Dr. Mitral” *Learners request consult and describe case* “Sounds like peripartum cardiomyopathy; please start inotropic support. We can evaluate her for mechanical circulatory support should she continue to worsen. Have you called Ob/Gyn?”			
15:00 (Case Complete)	Disposition to OR and ICU	The patient should be admitted to the intensive care unit for continued management. The obstetrics consult may suggest direct transfer to the operating room (OR) for emergent Cesarian section.	

**Figure f6-jetem-8-2-s1:**
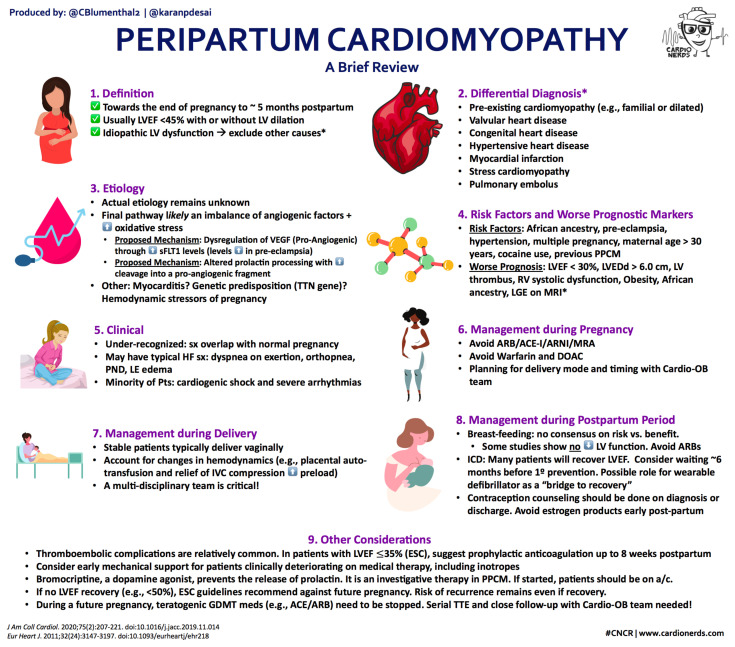
Reprinted with permission from the CardioNerds, from the following source: https://www.cardionerds.com/57-case-report-peripartumcardiomyopathy-with-cardiogenic-shock-university-of-pennsylvania/

**Table t9-jetem-8-2-s1:** 

Critical Actions:	
Connect the patient to the cardiac monitor and obtain a full set of initial vital signs Obtain IV access and laboratory studies Obtain history from patient, mother, and EMS Recognize and treat respiratory distress Evaluate fetal heart rate Obstetrics consult Recognize and treat cardiogenic shock Make the diagnosis of peripartum cardiomyopathy ICU admission	0:00

**Table t10-jetem-8-2-s1:** 

	Milestone	Did not achieve level 1	Level 1	Level 2	Level 3
1	**Emergency Stabilization (PC1)**	□ Did not achieve Level 1	□ Recognizes abnormal vital signs	□ Recognizes an unstable patient, requiring intervention Performs primary assessment Discerns data to formulate a diagnostic impression/plan	□ Manages and prioritizes critical actions in a critically ill patient Reassesses after implementing a stabilizing intervention
2	**Performance of focused history and physical (PC2)**	□ Did not achieve Level 1	□ Performs a reliable, comprehensive history and physical exam	□ Performs and communicates a focused history and physical exam based on chief complaint and urgent issues	□ Prioritizes essential components of history and physical exam given dynamic circumstances
3	**Diagnostic studies (PC3)**	□ Did not achieve Level 1	□ Determines the necessity of diagnostic studies	□ Orders appropriate diagnostic studies. Performs appropriate bedside diagnostic studies/procedures	□ Prioritizes essential testing Interprets results of diagnostic studies Reviews risks, benefits, contraindications, and alternatives to a diagnostic study or procedure
4	**Diagnosis (PC4)**	□ Did not achieve Level 1	□ Considers a list of potential diagnoses	□ Considers an appropriate list of potential diagnosis May or may not make correct diagnosis	□ Makes the appropriate diagnosis Considers other potential diagnoses, avoiding premature closure
5	**Pharmacotherapy (PC5)**	□ Did not achieve Level 1	□ Asks patient for drug allergies	□ Selects an medication for therapeutic intervention, consider potential adverse effects	□ Selects the most appropriate medication and understands mechanism of action, effect, and potential side effects Considers and recognizes drug-drug interactions
6	**Observation and reassessment (PC6)**	□ Did not achieve Level 1	□ Reevaluates patient at least one time during case	□ Reevaluates patient after most therapeutic interventions	□ Consistently evaluates the effectiveness of therapies at appropriate intervals
7	**Disposition (PC7)**	□ Did not achieve Level 1	□ Appropriately selects whether to admit or discharge the patient	□ Appropriately selects whether to admit or discharge Involves the expertise of some of the appropriate specialists	□ Educates the patient appropriately about their disposition Assigns patient to an appropriate level of care (ICU/Tele/Floor) Involves expertise of all appropriate specialists
9	**General Approach to Procedures (PC9)**	□ Did not achieve Level 1	□ Identifies pertinent anatomy and physiology for a procedure Uses appropriate Universal Precautions	□ Obtains informed consent Knows indications, contraindications, anatomic landmarks, equipment, anesthetic and procedural technique, and potential complications for common ED procedures	□ Determines a back-up strategy if initial attempts are unsuccessful Correctly interprets results of diagnostic procedure
20	**Professional Values (PROF1)**	□ Did not achieve Level 1	□ Demonstrates caring, honest behavior	□ Exhibits compassion, respect, sensitivity and responsiveness	□ Develops alternative care plans when patients’ personal beliefs and decisions preclude standard care
22	**Patient centered communication (ICS1)**	□ Did not achieve level 1	□ Establishes rapport and demonstrates empathy to patient (and family) Listens effectively	□ Elicits patient’s reason for seeking health care	□ Manages patient expectations in a manner that minimizes potential for stress, conflict, and misunderstanding. Effectively communicates with vulnerable populations, (at risk patients and families)
23	**Team management (ICS2)**	□ Did not achieve level 1	□ Recognizes other members of the patient care team during case (nurse, techs)	□ Communicates pertinent information to other healthcare colleagues	□ Communicates a clear, succinct, and appropriate handoff with specialists and other colleagues Communicates effectively with ancillary staff

**Table 1:**
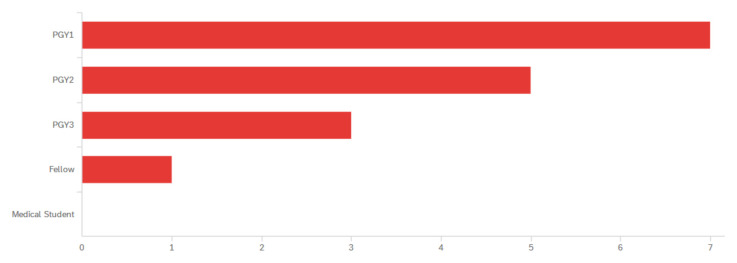
Identified levels of training for participants. Post Graduate Year (PGY).

**Table 2:** Post-Participation Feedback Survey

Question	Response
This simulation will improve my performance in an actual clinical setting.	Strongly Agree 81.25% Agree 18.75%
This simulation improved my ability to develop and prioritize evaluation and management options for this topic.	Strongly Agree 81.25% Agree 18.75%
The debriefing contributed to my understanding of the topic.	Strongly Agree 87.5% Agree 12.5%
The debriefing promoted reflection and team discussion.	Strongly Agree 81.25% Agree 18.75%
The facilitator created a safe environment for exploration and discussion.	Strongly Agree 81.25% Agree 18.75%
How could this experience be improved?	Free Response
Describe one way this simulation will change how you perform in the clinical setting.	Free Response

**Table 3:** Selected Survey Free Responses

*“Thought it was well done. Understanding the current hospital setting and what resources we have is helpful.”*
*“Excellent.”*
*“I’ll be more cognizant of managing the pregnant patient in cardiogenic shock, specifically regarding vasopressor choices.”*
*“Management of a rare but critically important case, best choice of [vasopressors], clear communication with consultants.”* *“Will probably not give start with phenylephrine as pressor in pregnancy, consider [venous] thromboembolism prophylaxis, consult [obstetrics] earlier.”*

## INSTRUCTOR MATERIALS

**Case Title:** Peripartum Cardiomyopathy

**Case Description & Diagnosis (short synopsis):** 36-year-old female G1P0 at 38 weeks gestation brought in by EMS after syncopal episode at home, presenting with three weeks of shortness of breath that acutely worsened today and is associated with lower extremity peripheral edema. The patient states she has been to two other emergency departments in the past three weeks where they have been dismissive of her symptoms, attributing her condition to normal pregnancy changes. Today the patient had a syncopal episode upon standing. The event was witnessed by her mother. It lasted a few seconds. The learner(s) should perform a primary survey to assess the patient. The learner(s) should recognize respiratory distress and initiate appropriate interventions. The goal of this case is for recognition of peripartum cardiomyopathy that can mimic other causes of hypotension and respiratory failure during pregnancy. During the case, the patient will decompensate and require ICU admission for cardiogenic shock. Cardiology and obstetrics consultations should be obtained.

**Equipment or Props Needed:**

High fidelity adult simulator/mannequin, pregnant mannequin preferred

Cardiac monitor with pulse oximetry

Blood pressure cuff

Intravenous (IV) bags and tubing

Prop medications – IV Syringes, IV Bags

- Etomidate
- Rocuronium
- Epinephrine
- Atropine
- Norepinephrine
- Dobutamine
- Digoxin
- Versed drip
- Fentanyl drip
- Ketamine

Airway Adjuncts

- Nasal cannula
- Non-rebreather mask
- Bag-valve-mask
- Positive pressure mask
- Video Laryngoscope
- Direct Laryngoscope
- Endotracheal tube with stylet
- Gum elastic bougie
- Laryngeal mask airway
- Ventilator
- Crash cart with defibrillator

IV Supplies

- Angiocatheter
- IV tubing
- Crystalloid

**Confederates needed:**

Roles can be played by a single operator or multiple persons as available. Simple clothing nametags or costumes may aid in clarity of role.

1. Nurse to assist with bedside management of the patient
  - Goal is to move the case forward by identifying any oversights or impasses that the care team may experience
2. Telephone consultation: Obstetric specialist for operative consultation and Intensive Care Unit (ICU) admission
  - Goal is to provide investigative and interventional opportunities and monitoring opportunities outside of the emergency physician’s scope of practice
3. Telephone consultation: Cardiology specialist for cardiac imaging and ICU admission
  - Goal is to provide investigative and interventional opportunities outside of the emergency physician’s scope of practice
4. Patient’s mother
  - Goal is to provide context to the general health of her daughter (the patient) and the events of the past few weeks. The mother may also notice the condition of the patient when the physicians do not and may help to clue the physicians about the severity of her condition.
5. Prehospital provider
  - Goal is to provide a summary and analysis of the scene where and how the patient was transported to the hospital (ie, “EMS history”).

**Stimulus Inventory:** [Table t5-jetem-8-2-s1]

**Background and brief information:** The scenario takes place in a tertiary/quaternary emergency department. The patient is a 36-year-old gravid woman who has just had a syncopal episode.

**Initial presentation:** The patient has been brought in by ambulance after having a witnessed syncopal episode at home. She has been triaged by nursing as an emergency services index (ESI) level 2. She is the next chart in queue to be seen. She is sitting up in the stretcher, alert, and in moderate respiratory distress.

**How the scene unfolds:** The patient is a 36-year-old woman G1P0 at 38 weeks gestation, laying sitting up in the bed, concerned about her baby. She is alert and oriented, but she has some difficulty speaking due to shortness of breath. Learners should rapidly assess the patient’s airway, breathing, circulation, and for disability. She should be placed on full cardiopulmonary monitors and learners should obtain a full set of vital signs. She has tachycardia, tachypnea, hypotension, and decreased oxygen saturation. She does not demonstrate any neurologic deficit. She explains that she has been symptomatic for several weeks but friends, family, and healthcare providers have been attributing her symptoms to “normal pregnancy.” This is her first pregnancy, and she does not have any other experience to compare it to. Today, the patient had a syncopal episode while at home with her mother, who witnessed the episode. There was no head trauma. She lost consciousness for “a couple of seconds.”

Learners should obtain a full history and cardiovascular-, obstetric-, and neurologic-focused physical exam. They should also be sure to elicit history from the mother and the EMS providers. The patient has multiple abnormal physical exam findings: hypotension, tachypnea, hypoxia, peripheral edema, and pulmonary rales.

Learners are expected to evaluate for common causes of syncope in a young woman, including checking a point of care glucose and rapidly reviewing an ECG. In addition, they should be concerned about her abnormal vital signs and actively work to improve the patient’s hypoxia, work of breathing, and hypotension. They should place an intravenous (IV) line and draw laboratory studies which they feel are appropriate. They should administer supplementary oxygen for her hypoxia and work of breathing and are expected to begin fluid resuscitation with isotonic fluid administration for hypotension. Many learners are well-versed in point of care ultrasound, and this may be part of their early workup.

They should recognize the patient is gravid and perform a bedside assessment of the fetal heart rate.

Regardless of the medications the patient receives, she will become progressively more hypotensive and more poorly perfused, exhibiting cardiogenic shock requiring vasopressors and intensive care unit (ICU) admission. The learners will need to consult obstetrics for fetal monitoring early in her presentation given her complaint of syncope. The learners should be concerned for PPCM and perform a point of care ultrasound (POCUS) and/or order a formal cardiac ultrasound to evaluate for left ventricular (LV) function. When they realize that the LV function is depressed, they should convey this to the cardiology consultant and obstetric consultant and request an intensive care unit (ICU) admission.

Because the patient is in cardiogenic shock, she should receive an arterial line for adequate blood pressure monitoring and perhaps a central venous line for vasopressor administration. If the patient does not have appropriate blood pressure management, she should complain of abdominal pain and fetal heart rate should depress (ie, fetal decelerations), or she should complain of worsening dyspnea. If she receives a large volume of rapidly infused fluid, she should complain of worsening dyspnea.

Respiratory interventions should include bilevel positive airway pressure (BPAP) or endotracheal intubation to decrease preload and afterload.

**Critical actions:**

1. Connect the patient to the cardiac monitor and obtain a full set of initial vital signs
2. Obtain IV access and laboratory studies
3. Obtain history from patient, mother, and EMS
4. Recognize and treat respiratory distress
5. Evaluate fetal heart rate
6. Obstetrics consult
7. Recognize and treat cardiogenic shock
8. Make the diagnosis of peripartum cardiomyopathy
9. ICU admission

**Case Title:** Peripartum Cardiomyopathy

**Chief Complaint:** “I passed out”[Table t6-jetem-8-2-s1]

**General Appearance:** Young, gravid-appearing woman in respiratory distress

**Primary Survey:**

- **Airway:** patent
- **Breathing:** tachypneic, speaking in 3–4-word sentences, bilateral basilar crackles
- **Circulation:** tachycardic, 2+ peripheral pulses, symmetric, bilateral lower extremity pitting edema to shins, normal capillary refill
- **Disability:** No focal neurologic deficit, alert and oriented

**History:**

- **EMS History:** EMS was called to scene for witnessed syncopal episode at home. Per family, patient stood up, then passed out. She was unresponsive for about ten seconds. She did not hit her head. There was no reported seizure-like activity.
- **Patient History:** 36-year-old female G1P0 at 38 weeks presenting after a syncopal episode at home and has been complaining of severe dyspnea for the past three weeks. She has been evaluated twice in the past two weeks in an urgent care center and an emergency center respectively, having been told that these are expectant pregnancy symptoms. She has had worsening bilateral leg pain and swelling her entire third trimester.
- **Past medical history:** No chronic medical conditions, first pregnancy (G1P0), no abortions, no miscarriages, has had regular prenatal care and normal prenatal course to date
- **Past surgical history:** None
- **Patient’s medications:** Prenatal vitamins
- **Allergies:** No Known Drug Allergies
- **Social history:** Non-smoker, No alcohol, No illicit drug use
- **Family history:** Mother with diabetes

**Secondary Survey/Physical Examination:**

- **General appearance:** appears stated age; sitting up in hospital bed, appears in moderate respiratory distress (tachypnea, speaks slowly with 3–4-word sentences, reports feeling uncomfortable)
- **HEENT:**
  ○ **Head:** Atraumatic, normocephalic
  ○ **Eyes:** Pupils equal, round, and reactive to light (4–2mm), external ocular movements are normal, normal conjunctiva, no papilledema
  ○ **Ears:** Normal
  ○ **Nose:** Normal
  ○ **Throat:** No erythema or edema of the oropharynx
  ○ **Neck:** Trachea midline, no stridor, jugular vein distention (JVD present
- **Heart:** Tachycardia, regular rhythm, equal pulses.
- **Lungs:** Moderate respiratory distress, tachypneic, speaks 3–4-word sentences, bibasilar crackles on auscultation
- **Abdominal/GI:** Soft, nontender, bowel sounds present, gravid with fundus above the umbilicus
- **Genitourinary:** Normal external GU, no vaginal bleeding, internal exam deferred
- **Rectal:** Normal
- **Extremities:** 2+ pitting edema in bilateral lower extremities extending to knees, no tenderness, no deformity, tolerates full range of motion, negative Homan’s sign bilaterally
- **Back:** Normal
- **Neuro:** Alert, oriented, normal reflexes, no clonus
- **Skin:** Cool, pale
- **Lymph:** Normal
- **Psychiatric:** Anxious but cooperative

**Results:** [Table t7-jetem-8-2-s1]

## OPERATOR MATERIALS

[Table t8-jetem-8-2-s1] **Diagnosis:**

Peripartum cardiomyopathy (PPCM), respiratory distress, cardiogenic shock

**Disposition:**

Admission to the intensive care unit or to the operating room.

## DEBRIEFING AND EVALUATION PEARLS

### Peripartum Cardiomyopathy

**Syncope** is the sudden and transient loss of consciousness and tone followed by spontaneous recovery. It is a clinical indicator of compromise of cerebral perfusion.

It is imperative that an emergency physician understands when to initiate an extensive evaluation in the ED versus a limited evaluation of the patient who presents with complaint of syncope.

- A detailed and contextual **history** is paramount when the chief complaint is syncope and should be obtained from any available witness.^6^
- An **ECG** should be obtained on any patient presenting with syncope to screen for causes of cardiogenic syncope. 6
- Any patient with abnormal vital signs and complaint of syncope is high-risk and requires further medical evaluation.

The history, physical, and ECG will then dictate the indicated laboratory tests and imaging tests which can include evaluations for most common causes of syncope. Particularly for causes of syncope in pregnancy:

- Amniotic fluid embolism
- Aortic dissection
- Aortocaval compression
- Arrhythmia
- Eclampsia
- Hemorrhage
- Hypoglycemia
- Orthostatic hypotension
- Peripartum cardiomyopathy
- Pulmonary embolism
- Vasovagal syncope

*This patient has a concerning history of present illness, is in late-stage pregnancy, has abnormal vital signs, and has an abnormal physical exam, which makes her complaint of syncope highly concerning*.

**Peripartum Cardiomyopathy (PPCM)**

**Definition:** idiopathic cardiomyopathy with new diagnosis of heart failure due to left ventricular (LV) systolic dysfunction during the last month of pregnancy or up to five months postpartum

- Diagnosis of exclusion
- The LV may not be dilated, but the ejection fraction is almost always reduced (<45%)

**Pathophysiology:**

- Definitive pathophysiology is uncertain, likely multifactorial
- Genetic predisposition
- Current theory suggests that timing suggests that hormonal changes (prolactin, tyrosine kinase) optimized at late pregnancy or early postpartum causes vascular insults

**Risk Factors:** woman 30+ years old, black woman, preeclampsia or hypertension, multiparous, malnourishment, smoking

- Shared risk factors with preeclampsia/eclampsia

**Clinical Features:**

- Typical heart failure symptoms: dyspnea, peripheral edema, orthopnea, etc.
- Rare: cardiogenic shock, arrhythmias

**Differential Diagnosis:** pulmonary embolism, amniotic fluid embolism, myocarditis, spontaneous coronary artery dissection (SCAD), acute coronary syndrome (ACS), Takotsubo cardiomyopathy, preeclampsia, normal pregnancy

**Laboratory Studies:** Nondiagnostic, but may help delineate from other differential diagnoses

- Brain natriuretic peptide (BNP), troponin – may be elevated to suggest congestive disease
- D-dimer is often commonly elevated in a normal pregnancy, so is less likely to be helpful
- Complete Blood Count (CBC), Liver Function Tests (LFTs), Lactate Dehydrogenase (LDH), uric acid, and fibrinogen – will help evaluate for preeclampsia
- Coagulation markers (PT/PTT/INR) – may be helpful to risk stratify because of increased risk for thromboembolic events

**Imaging Findings:**

- Chest x-ray: cardiomegaly, pulmonary congestion
- Echocardiogram: LV ejection fraction < 45%, often show right ventricle, left ventricle, or even atrial enlargement/dilatation, valvular function at the mitral and tricuspid may have some regurgitation, pulmonary hypertension is possible
- Electrocardiogram: sinus rhythm, sinus tachycardia, nonspecific T-wave or ST findings

**Treatment:**

- Airway & Breathing
  ○ Noninvasive positive pressure ventilation – decrease preload and afterload
  ○ Intubation – pregnant patients have higher risk for becoming hypoxic during rapid sequence intubation apnea due to low functional residual capacity and increased basal oxygen metabolic rate
- Circulation
  ○ Decrease preload – loop diuretics and nitrates
  ○ Decrease afterload – hydralazine or nitrates
  ○ Maintain cardiac output – vasopressors or inotropes; no consensus on best agent
- Thromboembolic prophylaxis
  ○ Hypercoagulable state intrinsically in peripartum period
  ○ Anticoagulation indicated when ejection fraction <30% and within 2–3 months following delivery^5^
  ○ Heparin and low molecular weight heparin are okay for use during pregnancy or breastfeeding
  ○ Warfarin and direct-acting oral anticoagulants (DOACs) should not be used during pregnancy for possible harm to fetus
- Pre-partum Considerations
  ○ Early continuous fetal heart monitoring
  ○ Decreasing volume status can compromise uterine perfusion
  ○ Avoid phenylephrine and norepinephrine which cause vasoconstriction of the uterine arteries which can cause placental insufficiency
  ○ Warfarin and DOACs should not be given
  ○ ACE inhibitors or ARBs should not be given to pregnant patients for possible injury to fetus
- Consultant Considerations
  ○ Obstetrics
    ▪ Emergency Cesarian section delivery
    ▪ Fetal heart rate monitoring
  ○ Cardiology
    ▪ Management of cardiogenic shock
    ▪ Cardiac assist devices (eg, LV assist device, extracorporeal membrane oxygenation (ECMO), intra-aortic balloon pump, cardiac defibrillator)

**Cardiogenic shock** is defined as failure of the pump mechanism of the heart to perfuse to the vital organs. Markers such as blood pressure or lactate help signify shock but are not intrinsically definitive.^7^ In the case of PPCM, the pump failure can be due to failure at the myocyte level causing difficulty with contractility and/or arrhythmia.

The goal mean arterial pressure (MAP) should be 65 or greater and should address the presumed cause of cardiogenic shock.

Because the major goal with PPCM cardiogenic shock is to increase contractility of the myocytes (inotropy) and ensure perfusion of the coronary arteries primarily, the medications of choice include dobutamine, norepinephrine, milrinone, and/or dopamine. No particular medication or combination of these agents is first line for PPCM.

Positive pressure ventilation is often necessary to assist with a patient with cardiogenic shock and respiratory distress. This will help with ventilation, oxygenation, and decrease the work of breathing for the patient. A typical sequela of positive pressure ventilation is decreased blood pressure by decreasing preload. This can be addressed by increasing the rate of inotropic or vasopressor infusion. The patient may also respond to a small isotonic fluid bolus.

Other considerations for cardiac support in shock should be for more invasive measures including cardiac assist devices, including LV assist devices, ECMO, intra-aortic balloon pump, and/or a cardiac defibrillator which can be navigated with the assistance of a cardiology consult.^7^

**Other debriefing points** **:**

If learners did not elicit from the EMS or patient’s parent, ask learners, “Were there any other sources of history you could have solicited?”

If learners did not intervene on the initial vital signs, ask them to analyze their thoughts on the initial vital signs. Interestingly, some residents described feeling more comfortable and had more experience identifying a patient *in extremis* when a geriatric patient or a patient with chronic illness. It may be useful to ask, “What does a patient *in extremis* look like?”

If learners do not place the patient on BPAP or did not start vasopressors/inotropes, ask learners, “How else could we support this patient with our skills and resources in the emergency department?”

If the learners are vague in their consultations, ask them, “What special skills or resources does this consultant have that can help this patient?”

**Wrap Up** **:** [Fig f6-jetem-8-2-s1]

The following terms and conditions apply as follows: Licensing material from an Elsevier journal: Reproduction limited to republication for this article.

## SIMULATION ASSESSMENT

*Peripartum Cardiomyopathy*

Learner: _________________________________________

***Assessment Timeline***

This timeline is to help observers assess their learners. It allows observer to make notes on when learners performed various tasks, which can help guide debriefing discussion.[Table t9-jetem-8-2-s1]

*Peripartum Cardiomyopathy*

Learner: _________________________________________

**Critical Actions:**

□ Connect the patient to the cardiac monitor and obtain a full set of initial vital signs
□ Obtain IV access and laboratory studies
□ Obtain history from patient, mother, and EMS
□ Recognize and treat respiratory distress
□ Evaluate fetal heart rate
□ Obstetrics consult
□ Recognize and treat cardiogenic shock
□ Make the diagnosis of peripartum cardiomyopathy
□ ICU admission

**Summative and formative comments:**

*Peripartum Cardiomyopathy*

Learner: _________________________________________

**Milestones assessment:** [Table t10-jetem-8-2-s1]
