# Supplementary material for: Peripartum Cardiomyopathy
Source: J Educ Teach Emerg Med. 2023 Apr 30;8(2):S1–S34. doi: 10.21980/J8ZS9M (PMC10332675; doi:10.21980/J8ZS9M)
Supplement: Supplementary file 1 [file JETem-8-2-S1-supp1.pptx]

## Slide 1
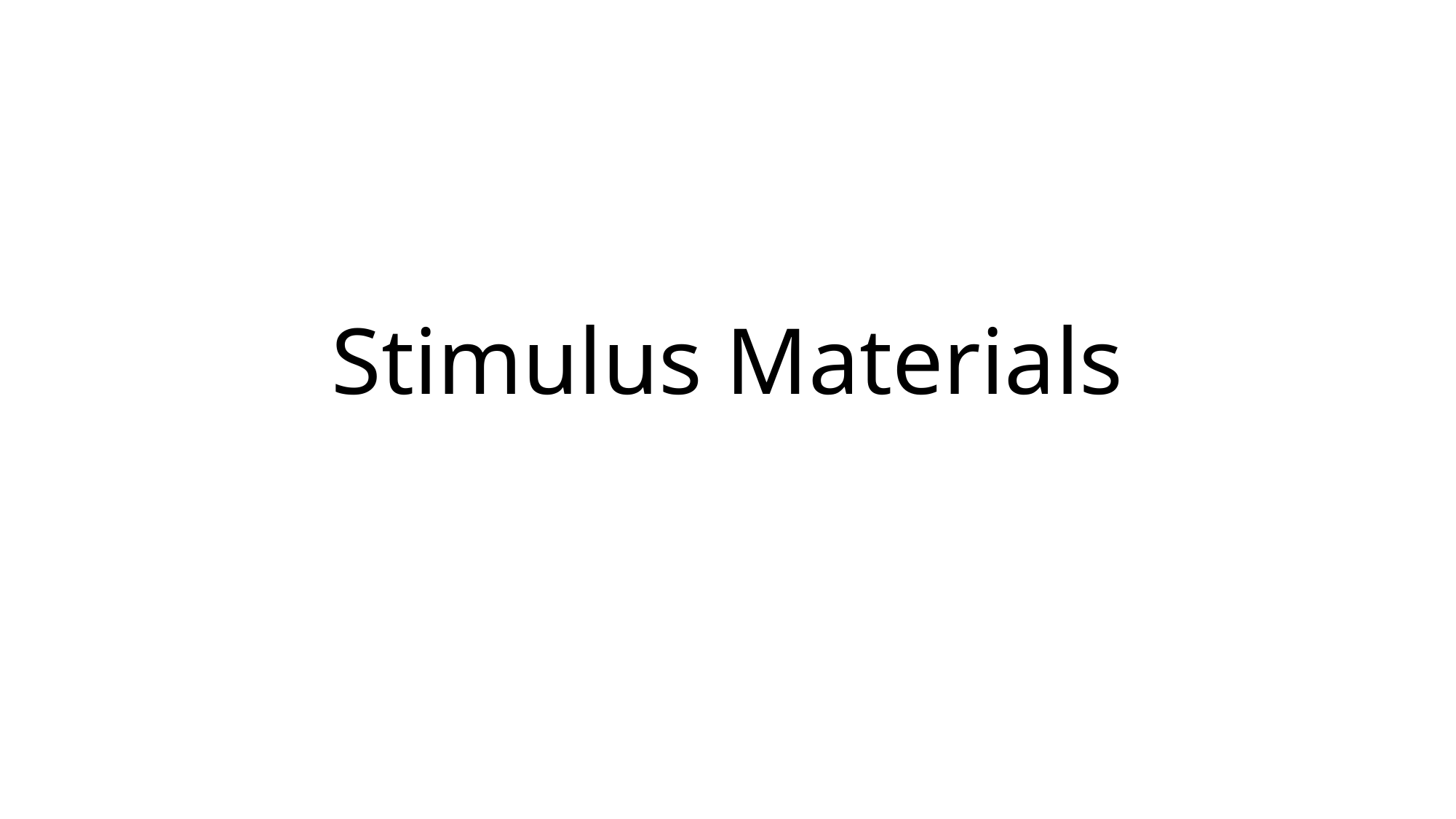

# Stimulus Materials

## Slide 2
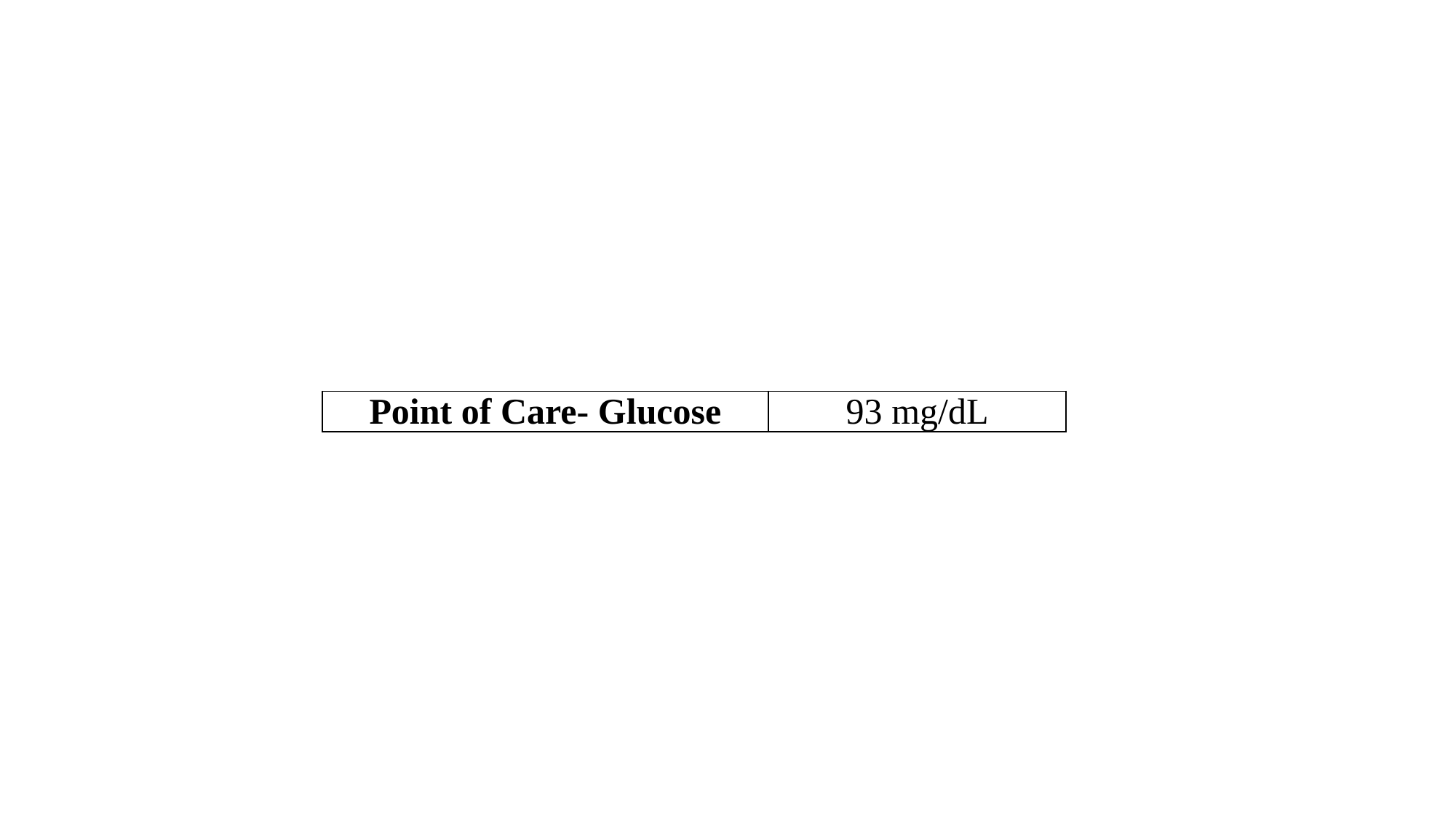

| Point of Care- Glucose | 93 mg/dL |
| --- | --- |

## Slide 3
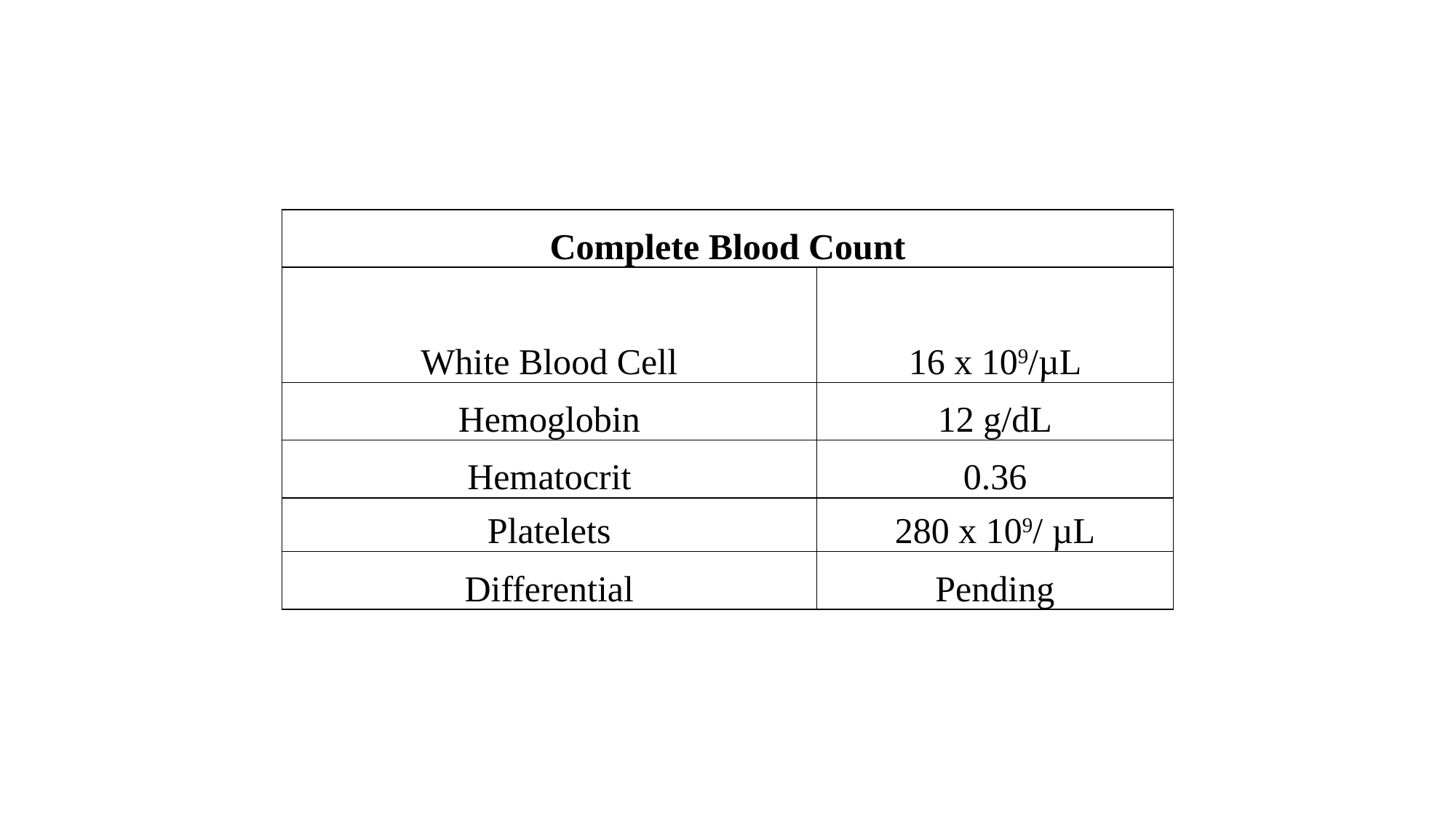

| Complete Blood Count | |
| --- | --- |
| White Blood Cell | 16 x 109/µL |
| Hemoglobin | 12 g/dL |
| Hematocrit | 0.36 |
| Platelets | 280 x 109/ µL |
| Differential | Pending |

## Slide 4
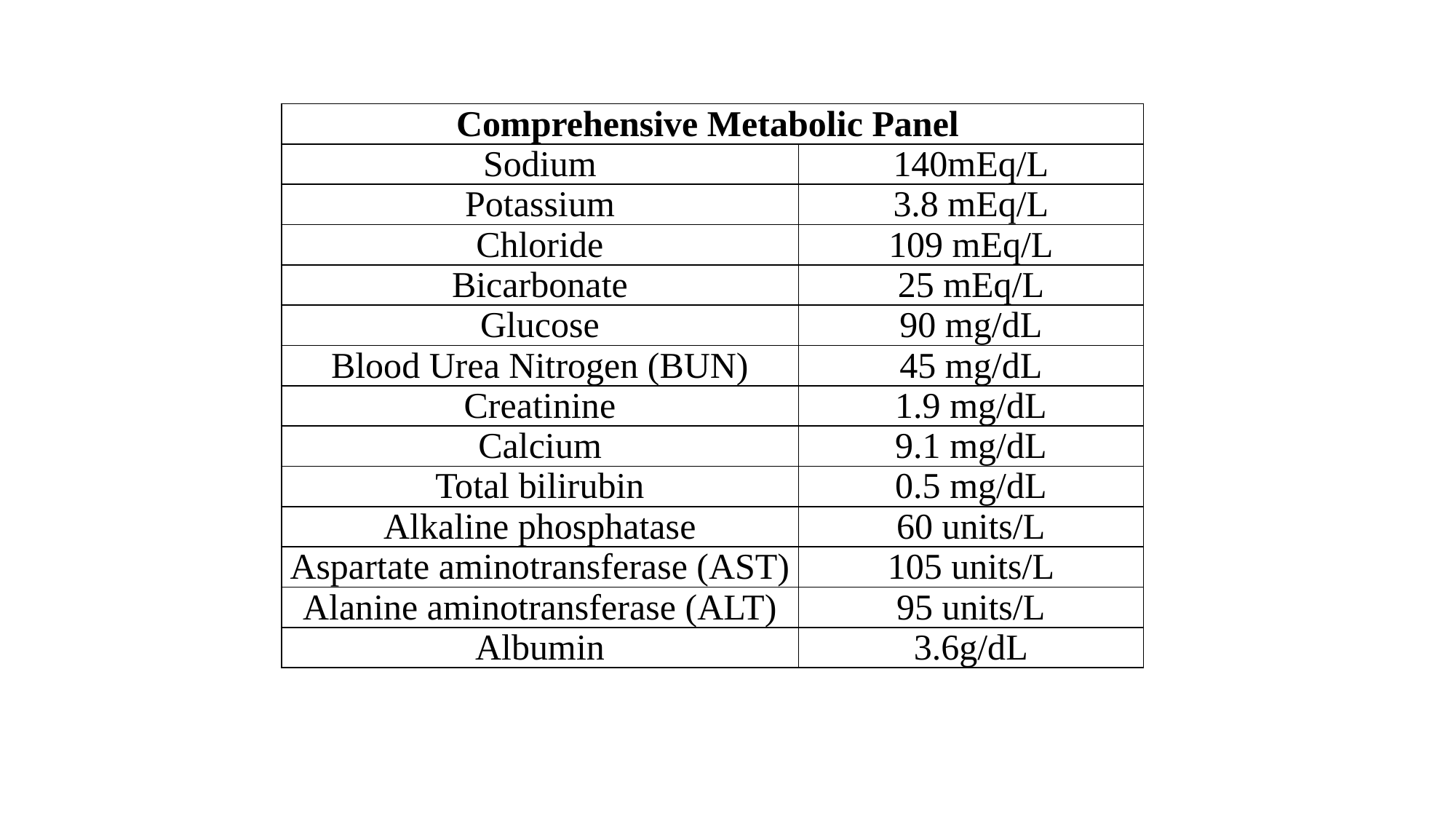

| Comprehensive Metabolic Panel | |
| --- | --- |
| Sodium | 140mEq/L |
| Potassium | 3.8 mEq/L |
| Chloride | 109 mEq/L |
| Bicarbonate | 25 mEq/L |
| Glucose | 90 mg/dL |
| Blood Urea Nitrogen (BUN) | 45 mg/dL |
| Creatinine | 1.9 mg/dL |
| Calcium | 9.1 mg/dL |
| Total bilirubin | 0.5 mg/dL |
| Alkaline phosphatase | 60 units/L |
| Aspartate aminotransferase (AST) | 105 units/L |
| Alanine aminotransferase (ALT) | 95 units/L |
| Albumin | 3.6g/dL |

## Slide 5
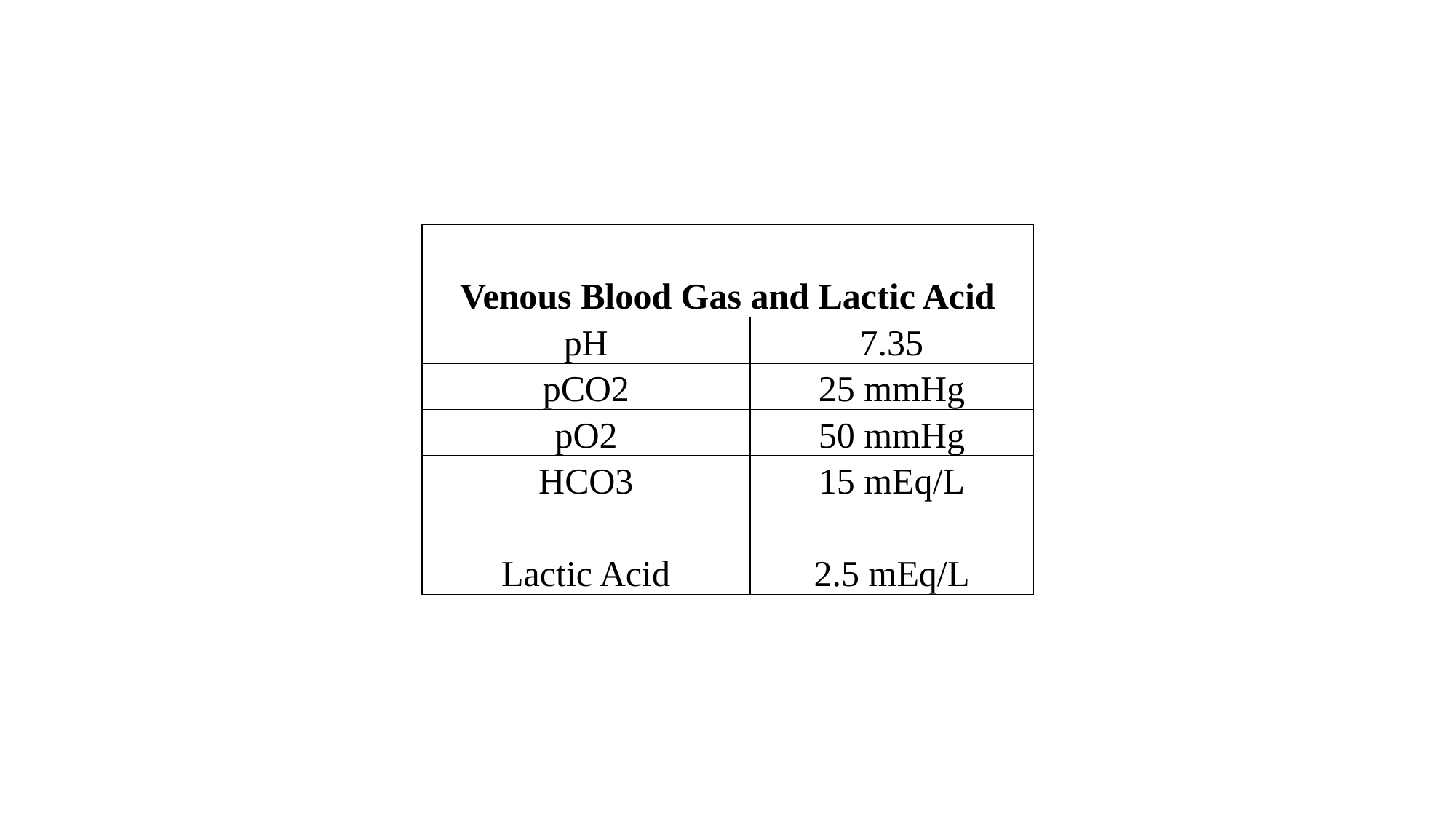

| Venous Blood Gas and Lactic Acid | |
| --- | --- |
| pH | 7.35 |
| pCO2 | 25 mmHg |
| pO2 | 50 mmHg |
| HCO3 | 15 mEq/L |
| Lactic Acid | 2.5 mEq/L |

## Slide 6
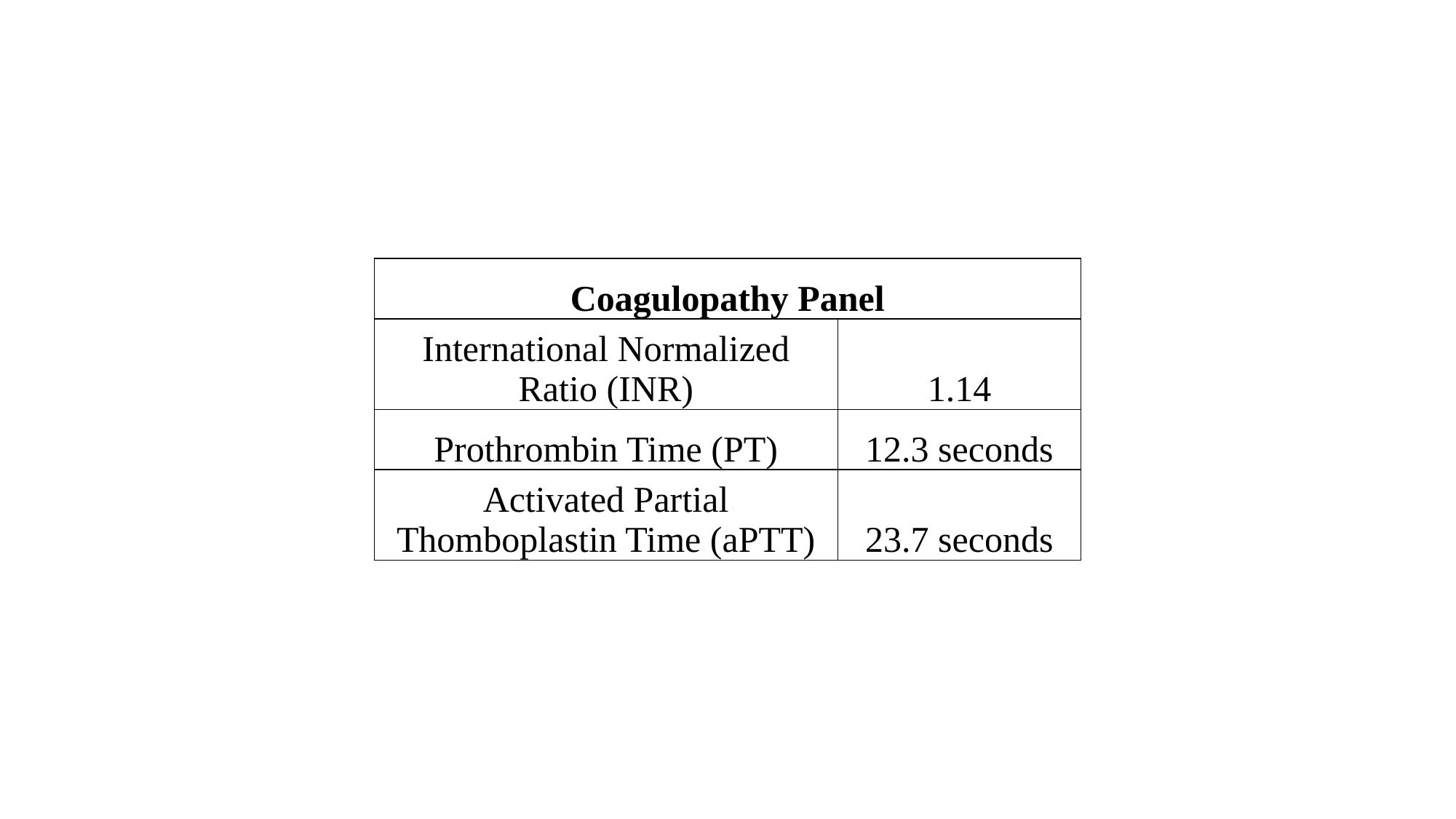

| Coagulopathy Panel | |
| --- | --- |
| International Normalized Ratio (INR) | 1.14 |
| Prothrombin Time (PT) | 12.3 seconds |
| Activated Partial Thomboplastin Time (aPTT) | 23.7 seconds |

## Slide 7
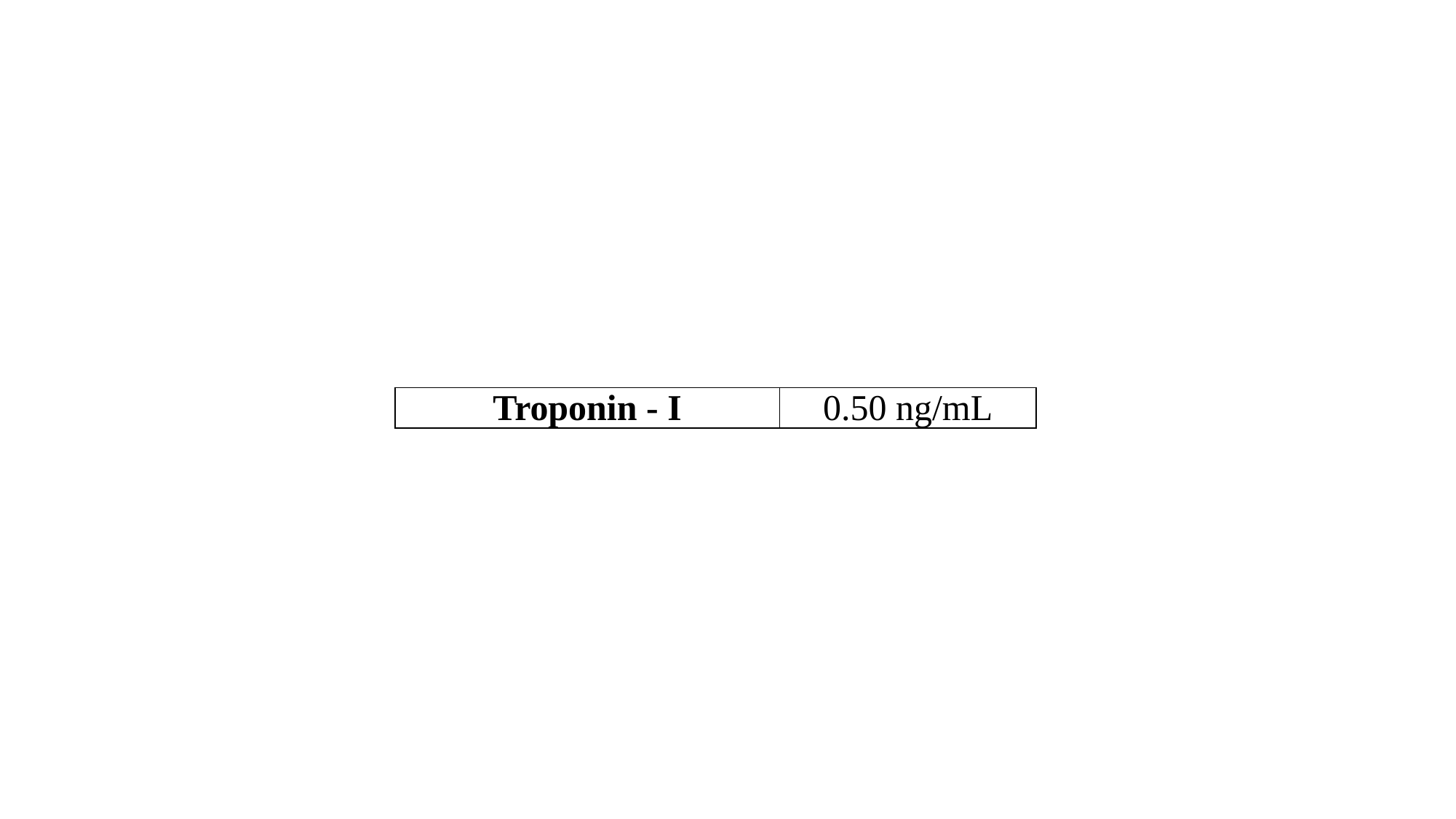

| Troponin - I | 0.50 ng/mL |
| --- | --- |

## Slide 8
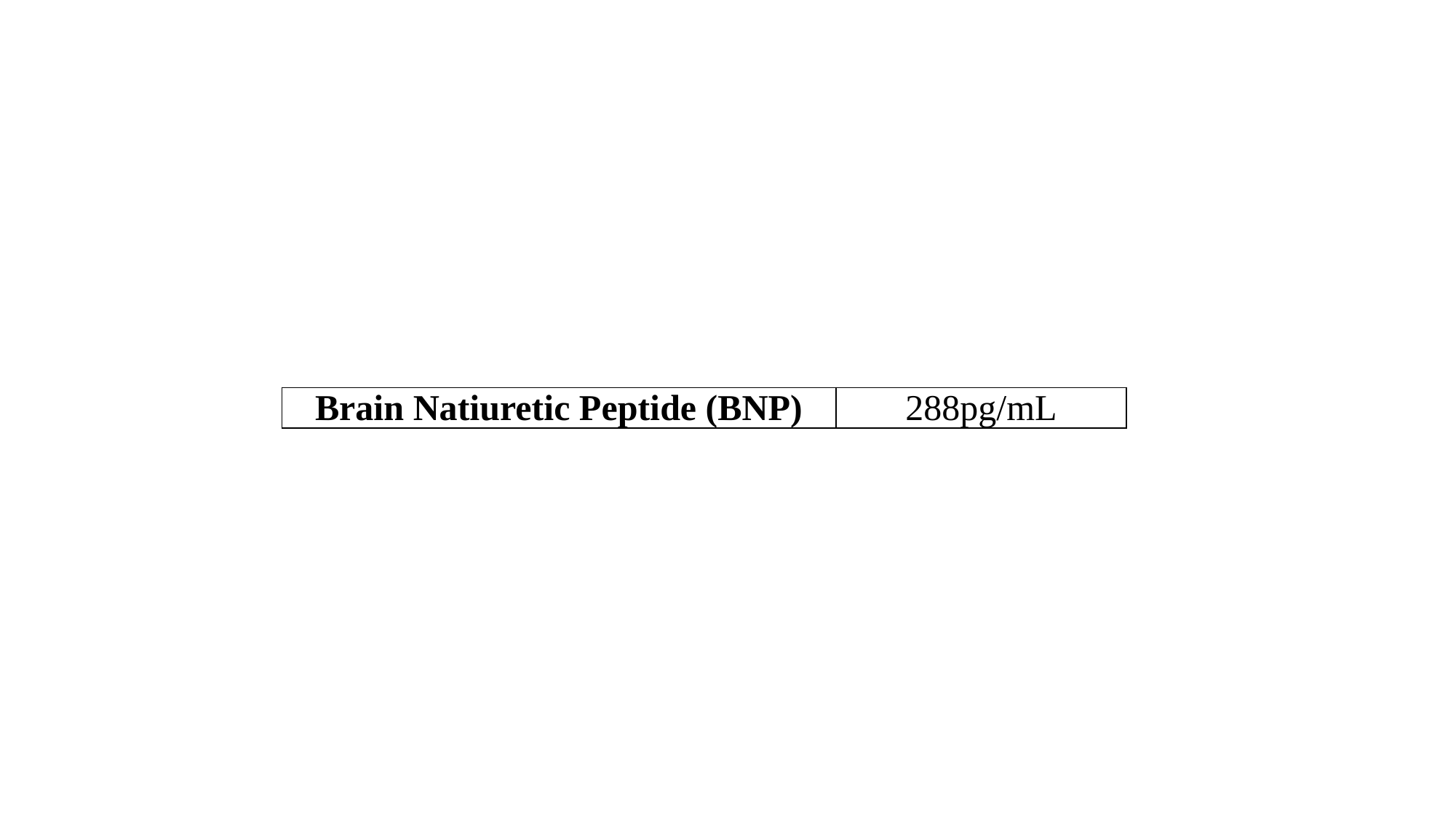

| Brain Natiuretic Peptide (BNP) | 288pg/mL |
| --- | --- |

## Slide 9
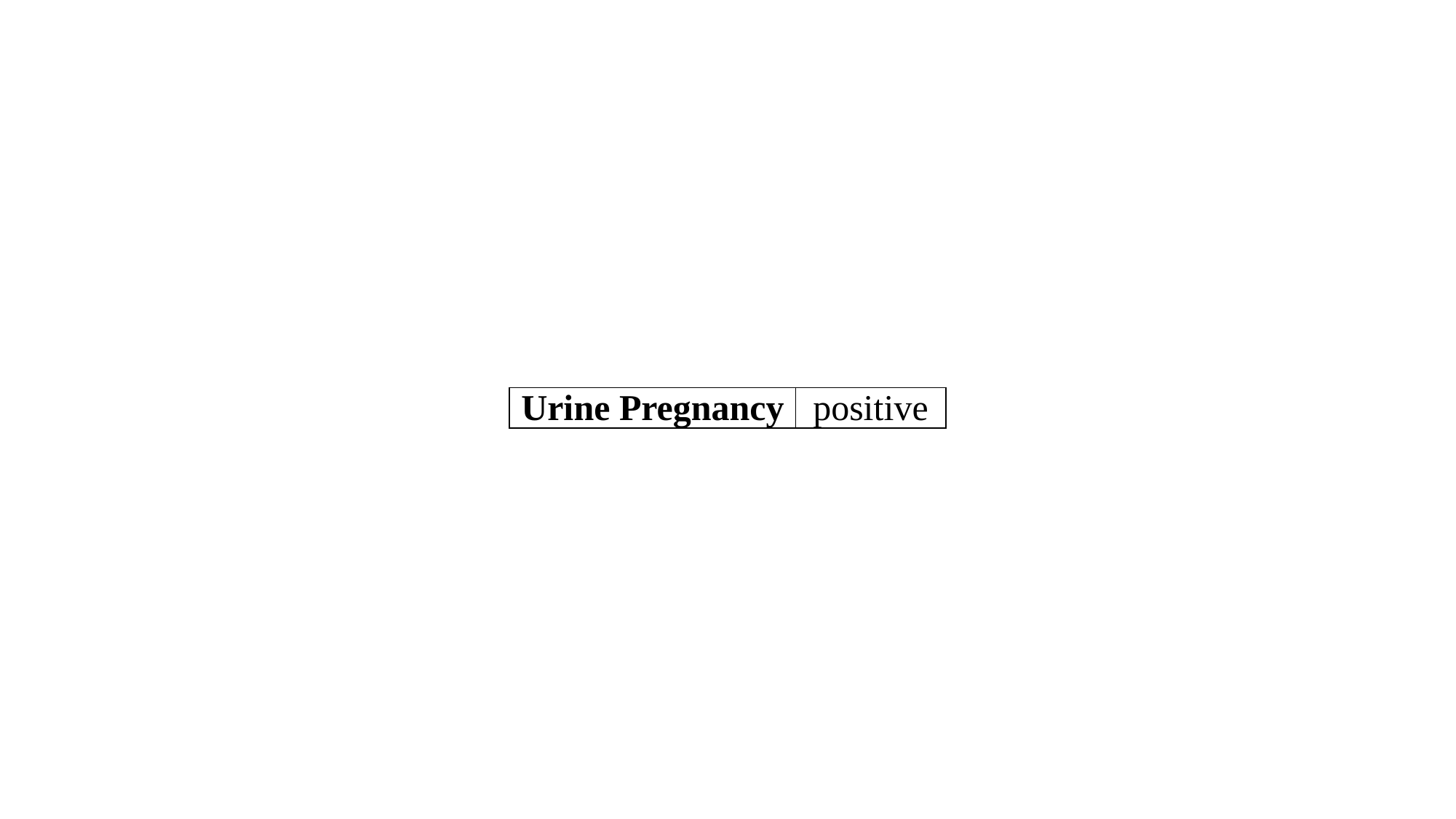

| Urine Pregnancy | positive |
| --- | --- |

## Slide 10
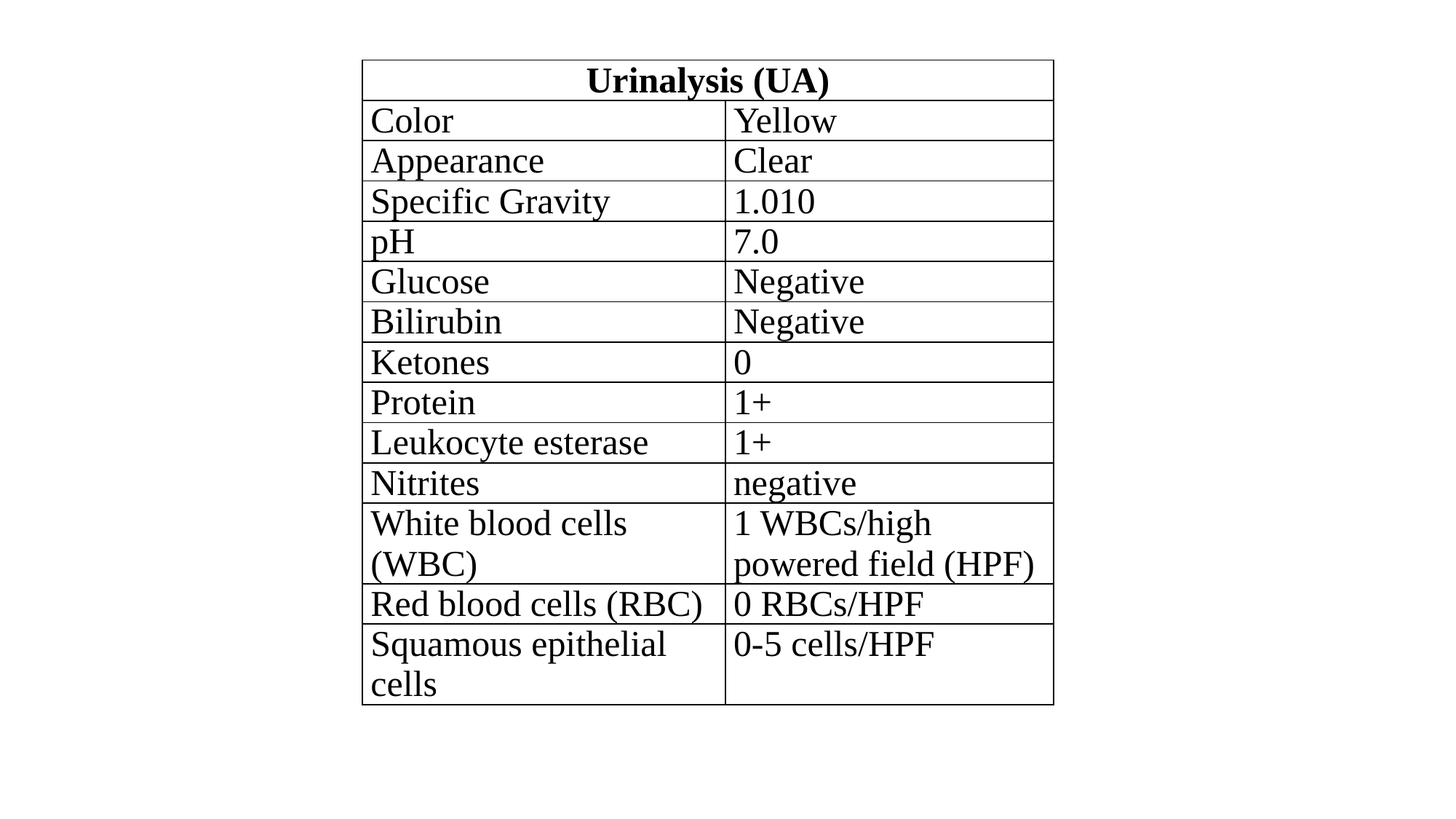

| Urinalysis (UA) | |
| --- | --- |
| Color | Yellow |
| Appearance | Clear |
| Specific Gravity | 1.010 |
| pH | 7.0 |
| Glucose | Negative |
| Bilirubin | Negative |
| Ketones | 0 |
| Protein | 1+ |
| Leukocyte esterase | 1+ |
| Nitrites | negative |
| White blood cells (WBC) | 1 WBCs/high powered field (HPF) |
| Red blood cells (RBC) | 0 RBCs/HPF |
| Squamous epithelial cells | 0-5 cells/HPF |

## Slide 11
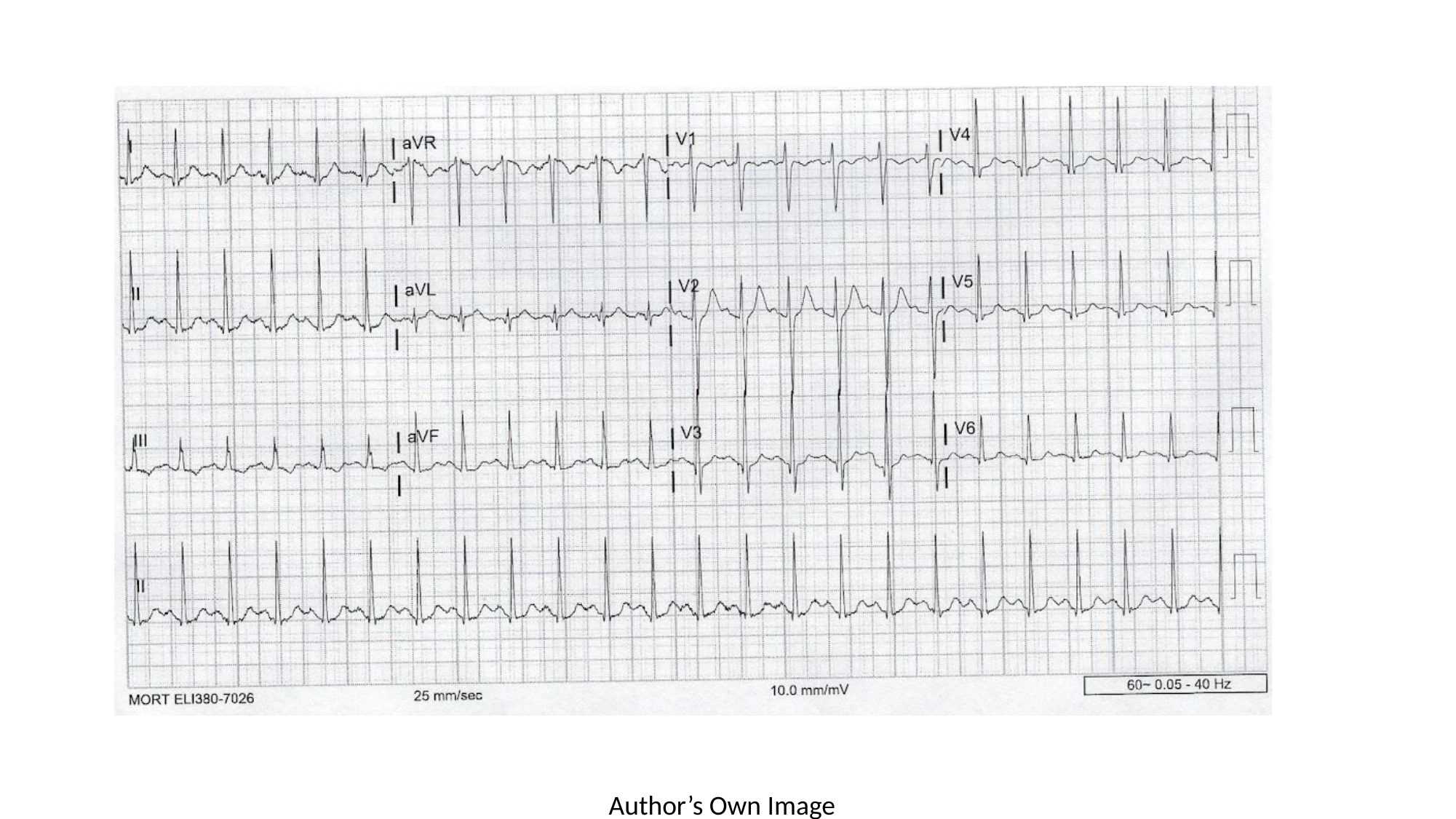

Author’s Own Image

## Slide 12
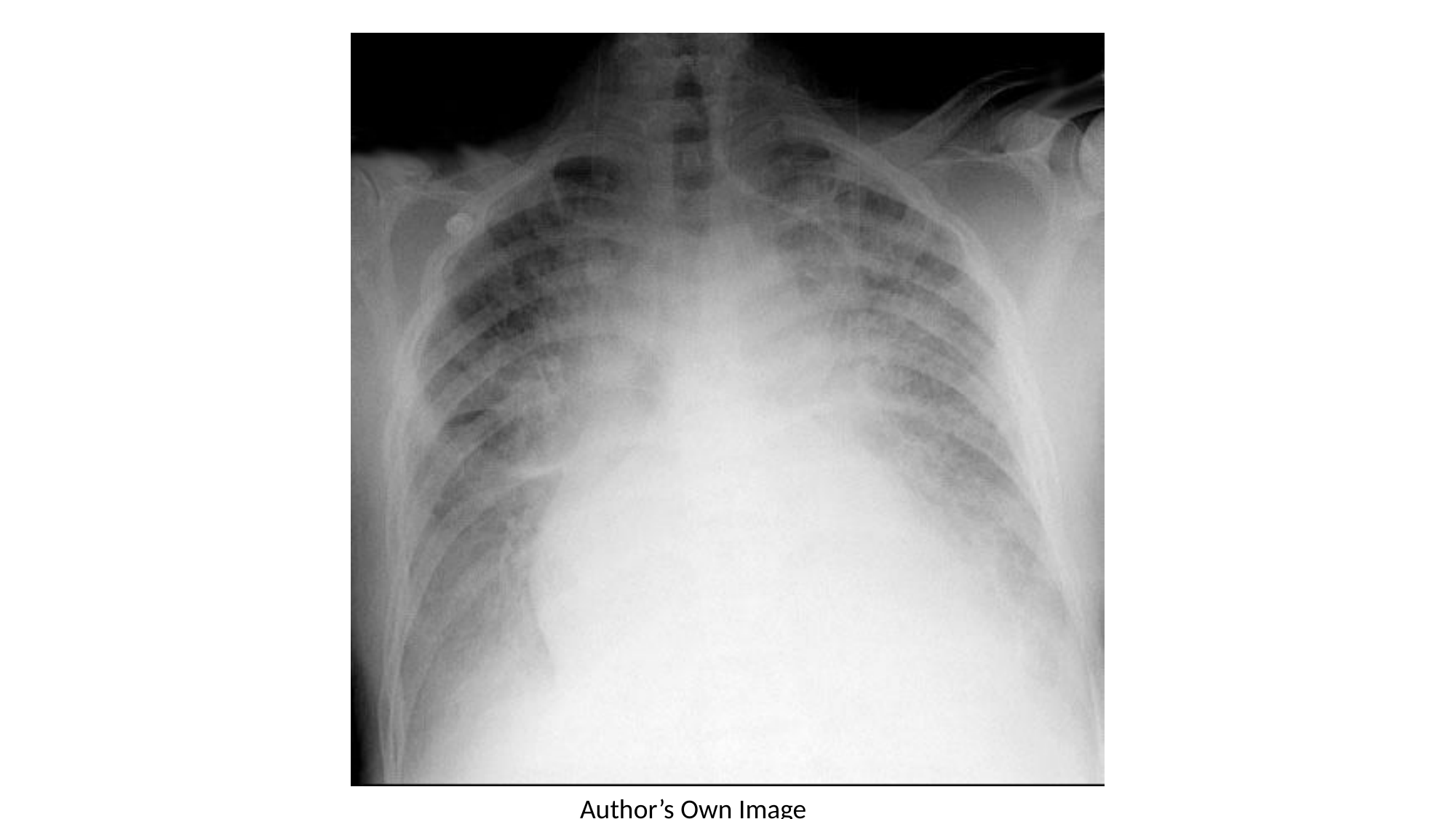

Author’s Own Image

## Slide 13
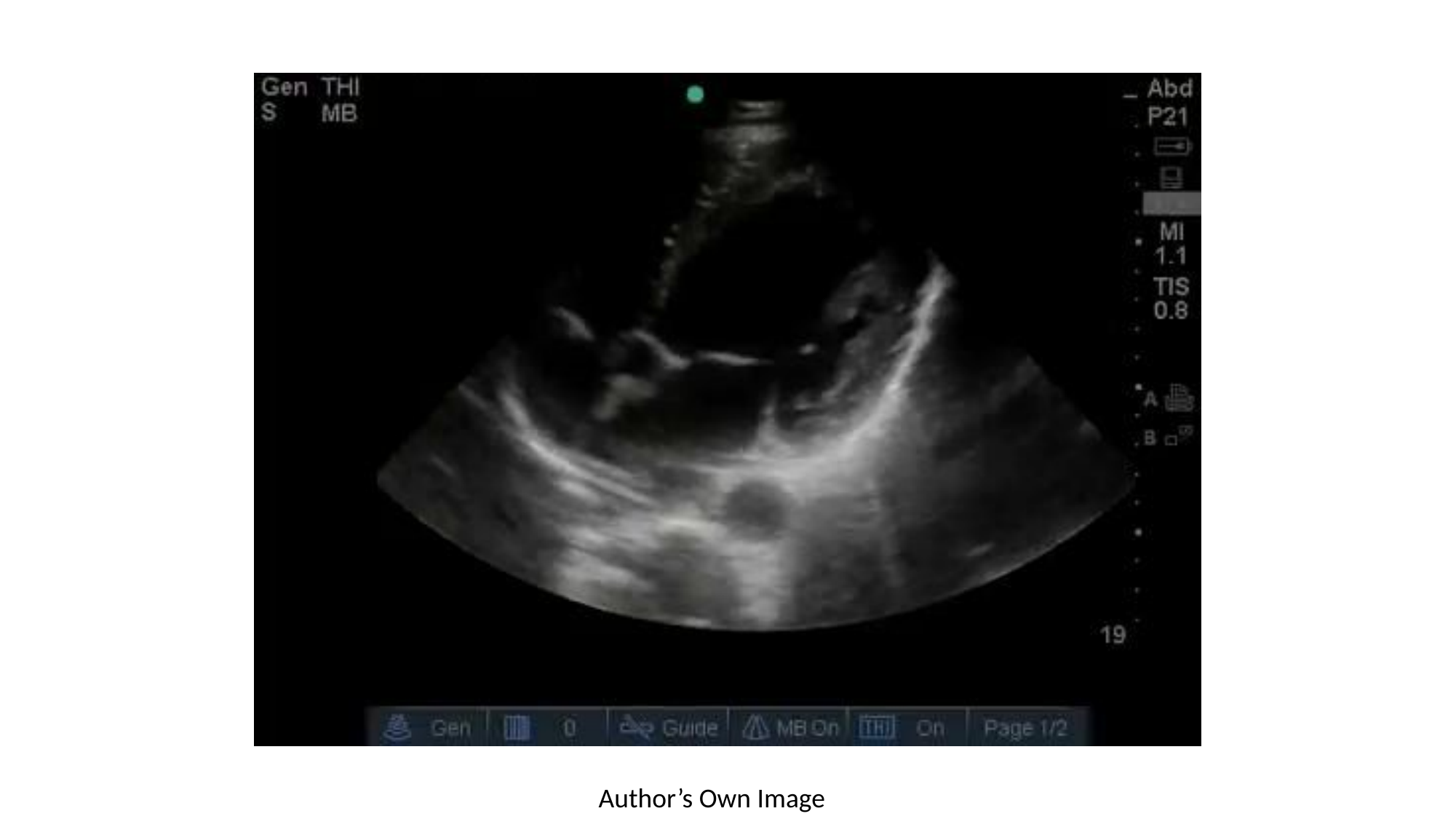

Author’s Own Image

## Slide 14
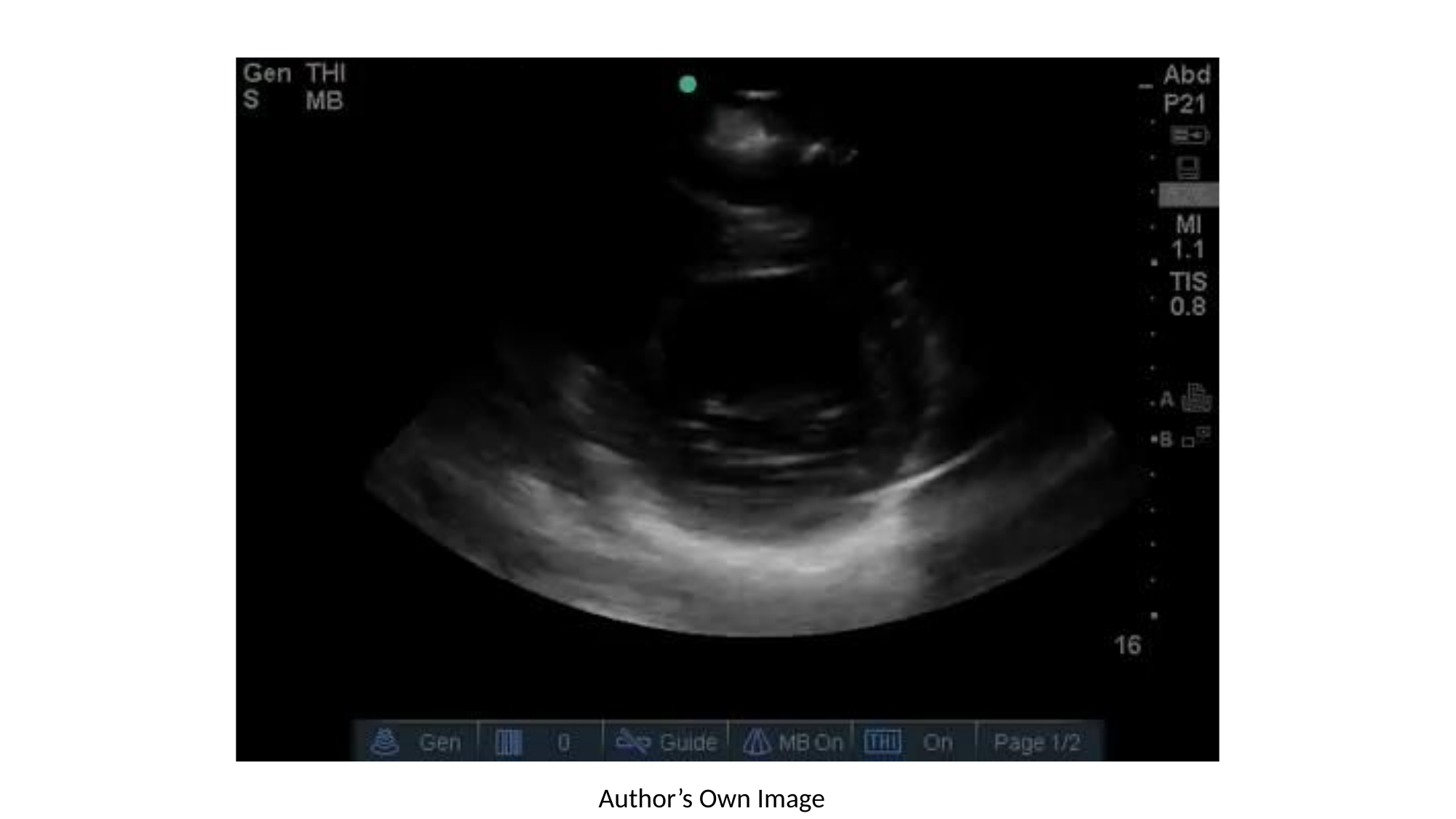

Author’s Own Image

## Slide 15
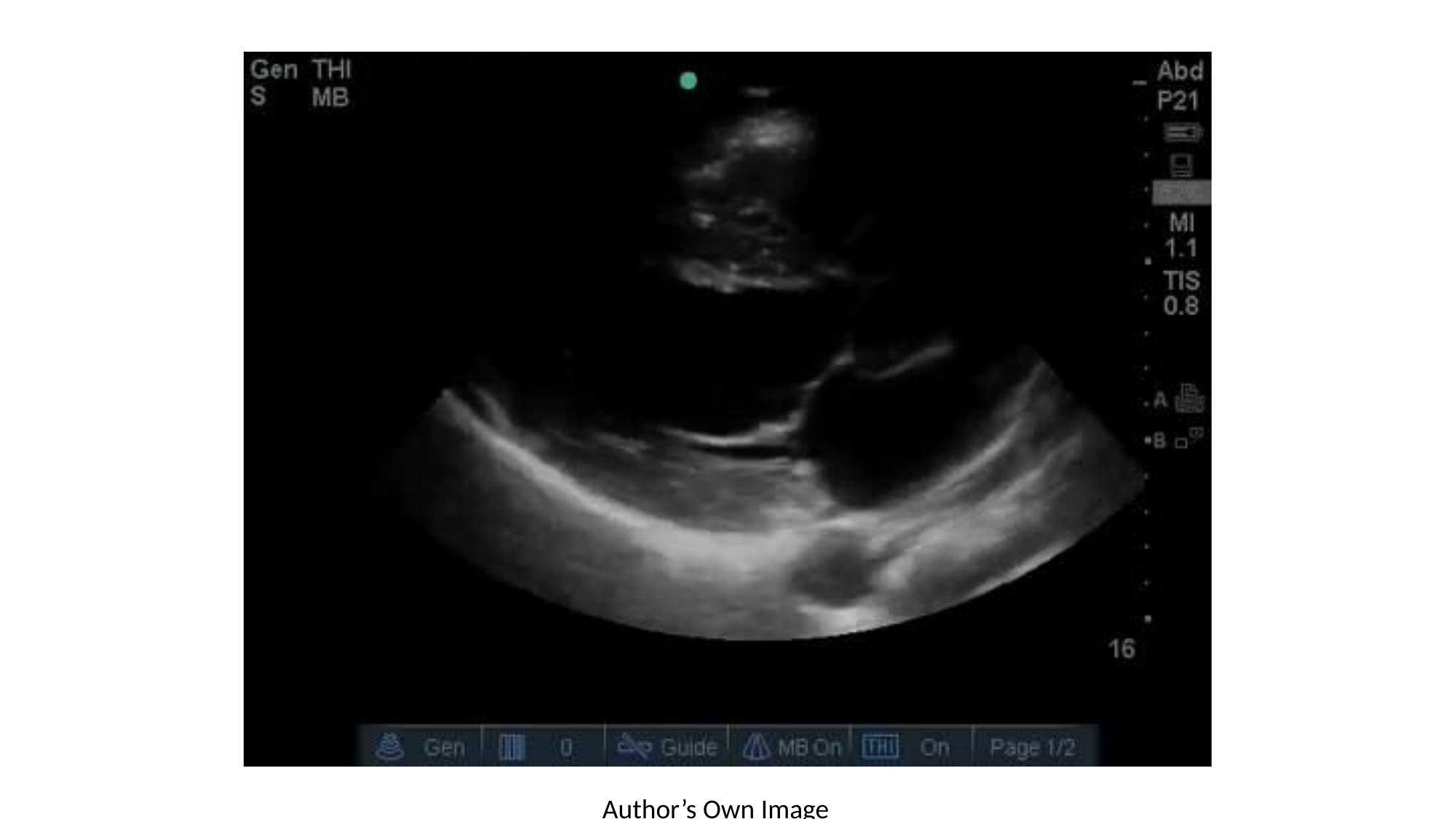

Author’s Own Image

## Slide 16
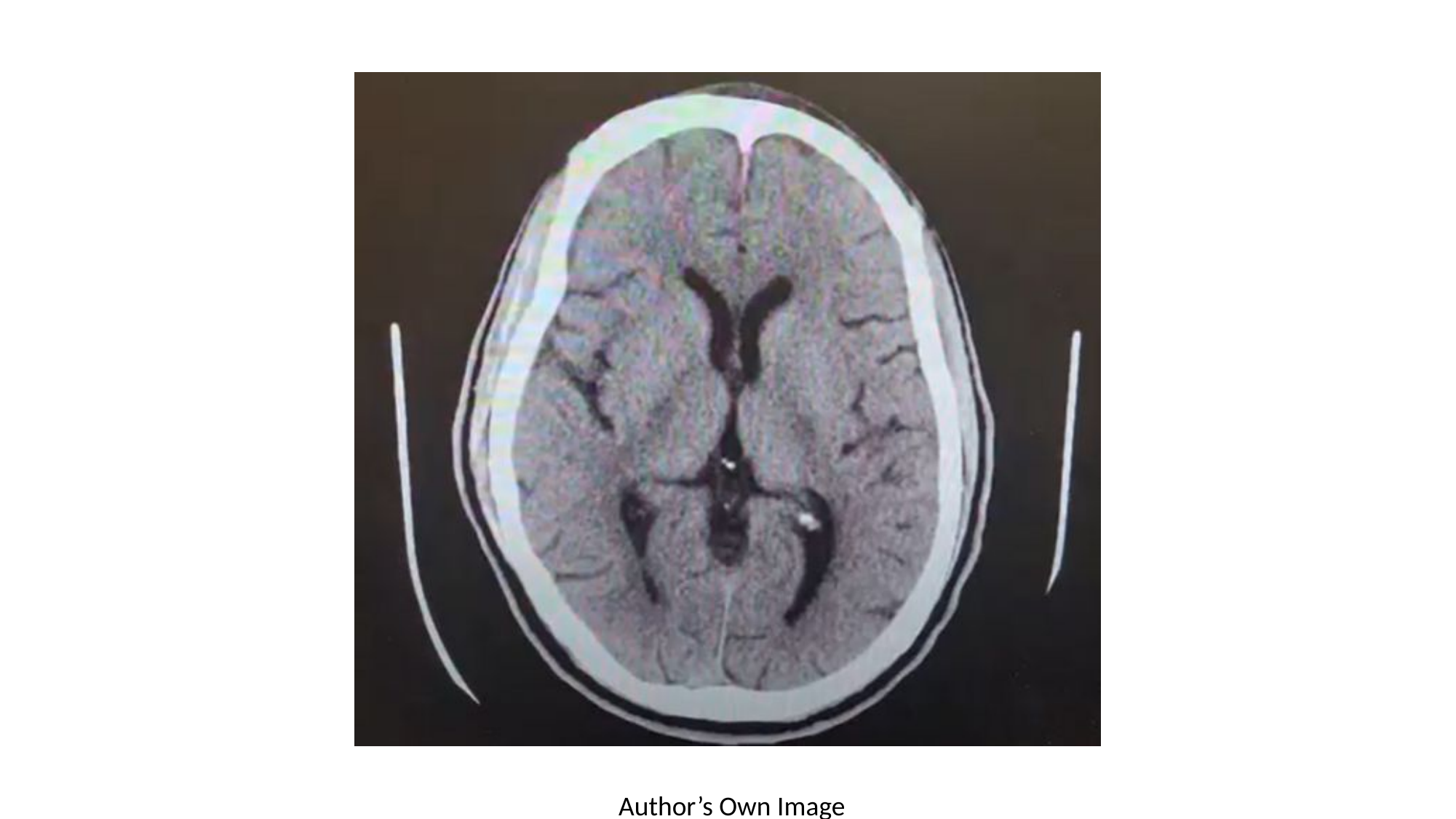

Author’s Own Image

## Slide 17
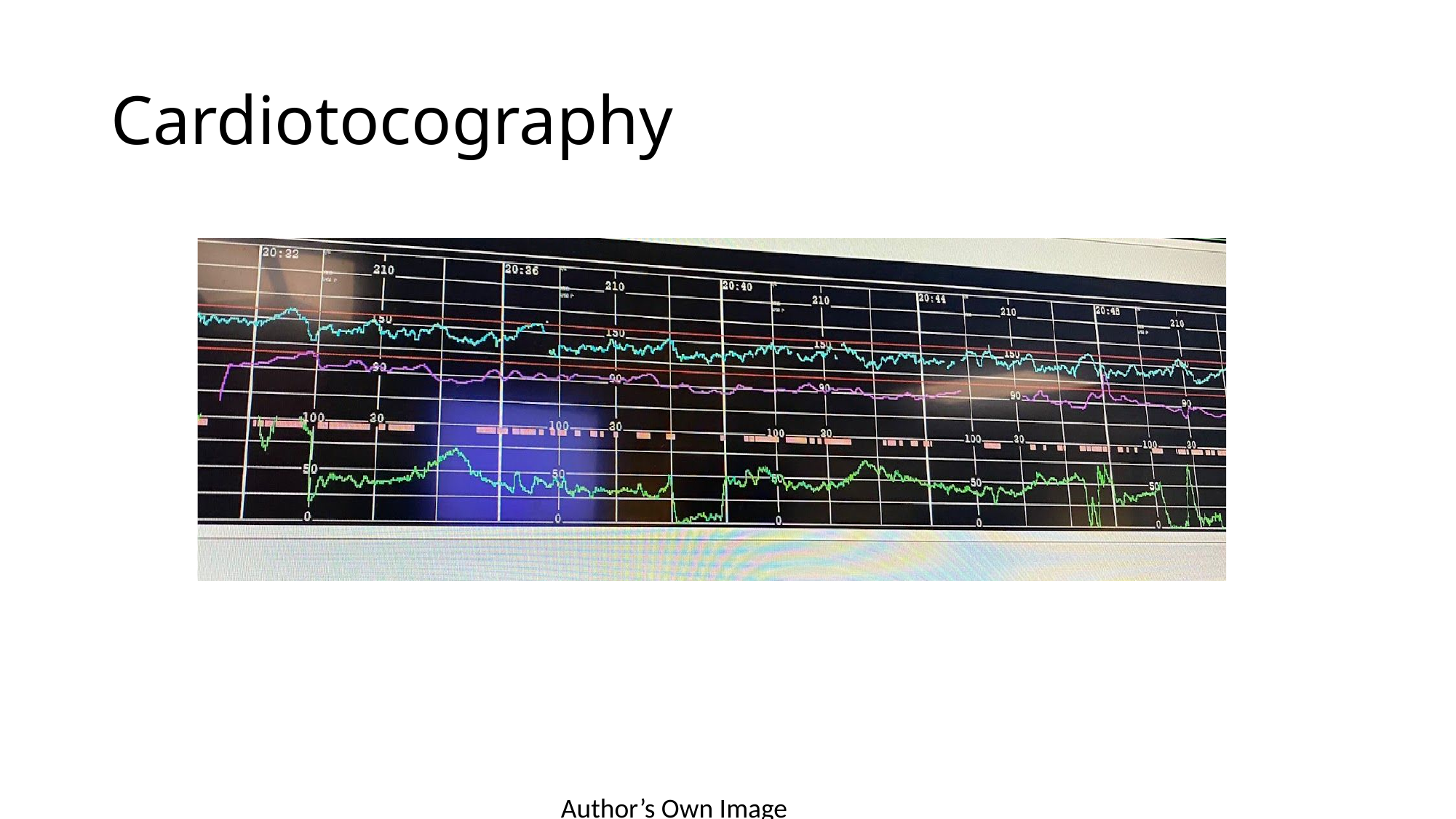

# Cardiotocography
Author’s Own Image
