# Supplementary material for: Peripartum Cardiomyopathy
Source: J Educ Teach Emerg Med. 2023 Apr 30;8(2):S1–S34. doi: 10.21980/J8ZS9M (PMC10332675; doi:10.21980/J8ZS9M)
Supplement: Supplementary file 2 [file JETem-8-2-S1-supp2.pptx]

## Slide 1
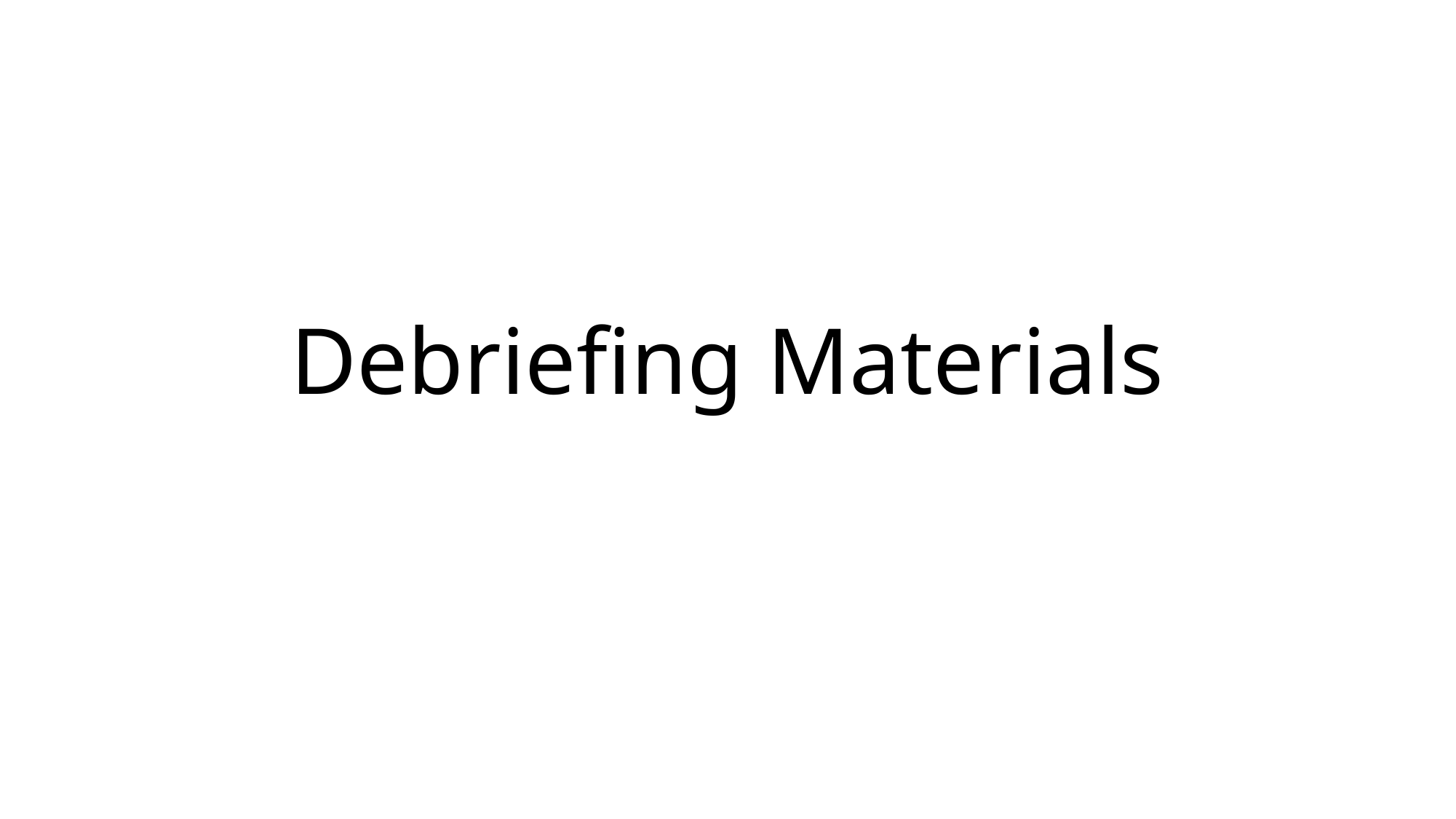

# Debriefing Materials

## Slide 2
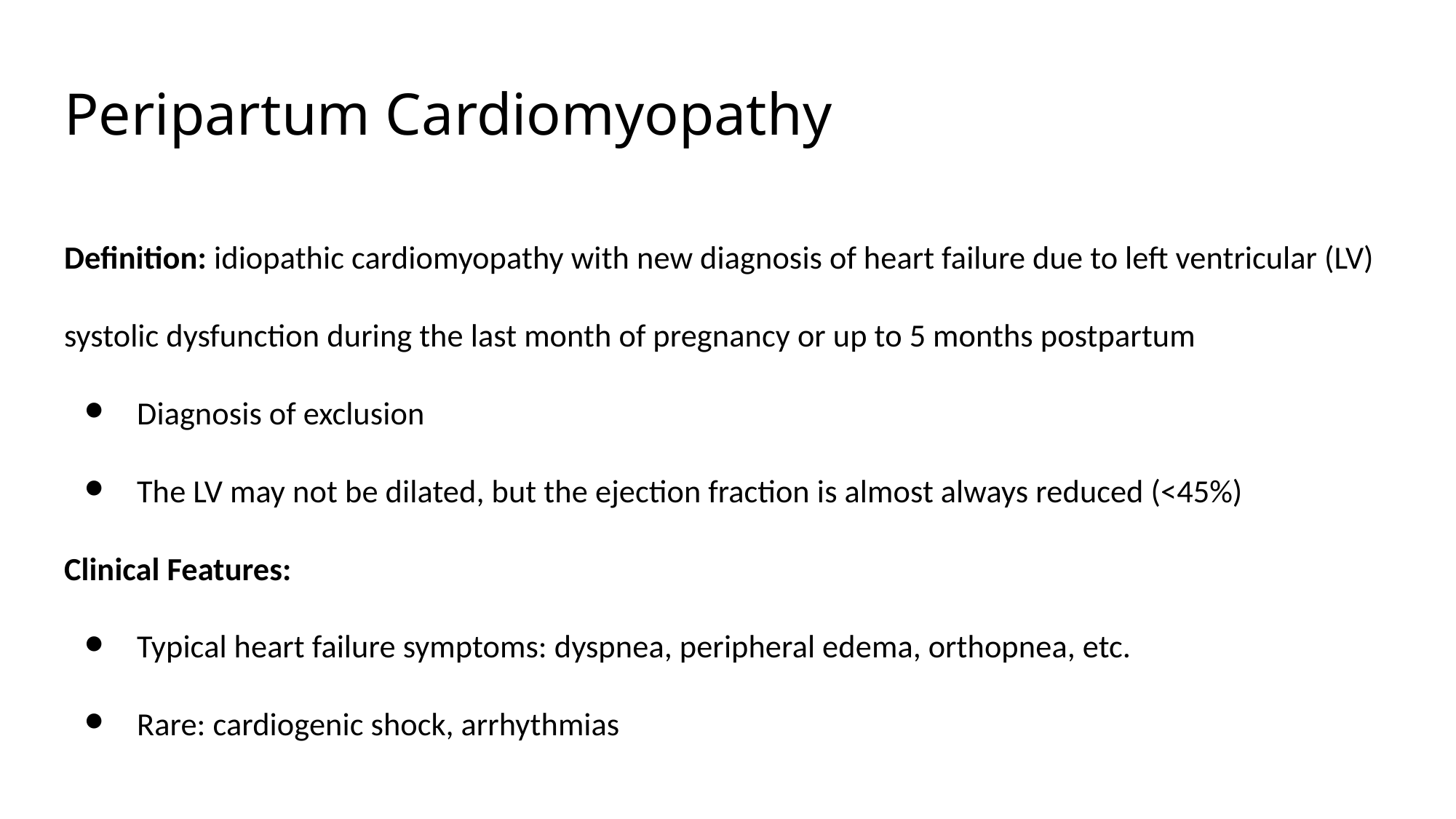

# Peripartum Cardiomyopathy
Definition: idiopathic cardiomyopathy with new diagnosis of heart failure due to left ventricular (LV) systolic dysfunction during the last month of pregnancy or up to 5 months postpartum
Diagnosis of exclusion
The LV may not be dilated, but the ejection fraction is almost always reduced (<45%)
Clinical Features:
Typical heart failure symptoms: dyspnea, peripheral edema, orthopnea, etc.
Rare: cardiogenic shock, arrhythmias

## Slide 3
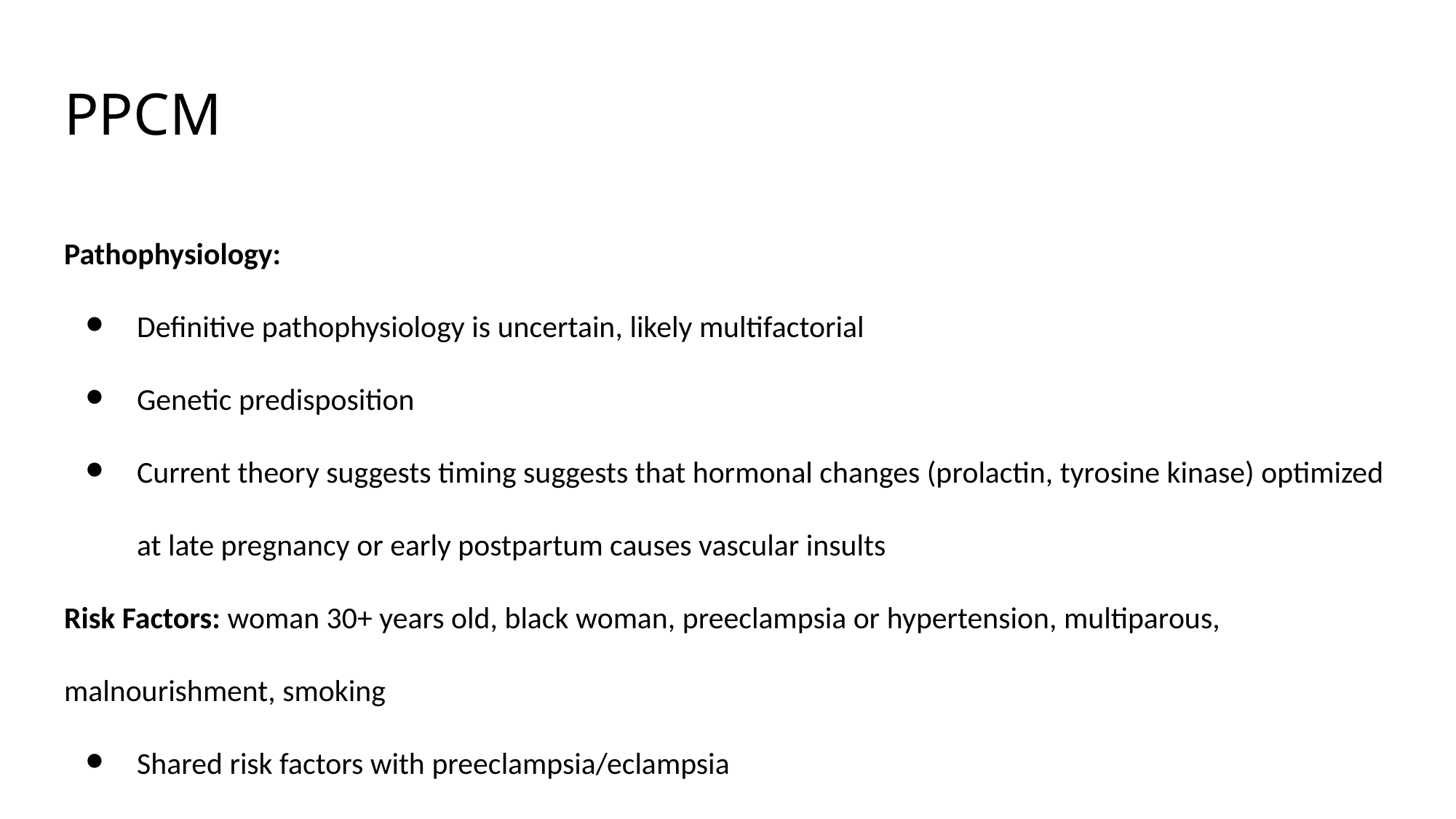

# PPCM
Pathophysiology:
Definitive pathophysiology is uncertain, likely multifactorial
Genetic predisposition
Current theory suggests timing suggests that hormonal changes (prolactin, tyrosine kinase) optimized at late pregnancy or early postpartum causes vascular insults
Risk Factors: woman 30+ years old, black woman, preeclampsia or hypertension, multiparous, malnourishment, smoking
Shared risk factors with preeclampsia/eclampsia

## Slide 4
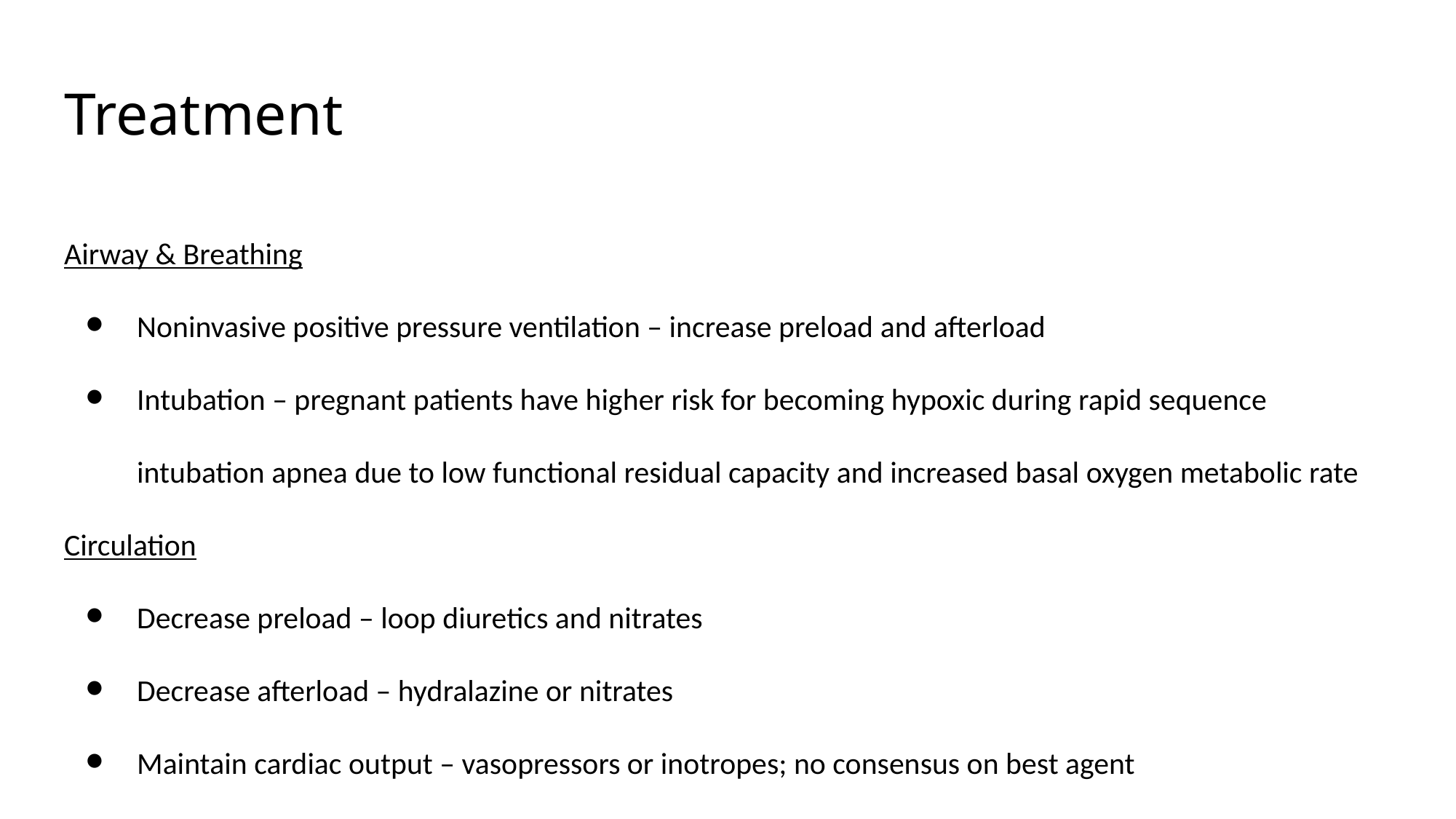

# Treatment
Airway & Breathing
Noninvasive positive pressure ventilation – increase preload and afterload
Intubation – pregnant patients have higher risk for becoming hypoxic during rapid sequence intubation apnea due to low functional residual capacity and increased basal oxygen metabolic rate
Circulation
Decrease preload – loop diuretics and nitrates
Decrease afterload – hydralazine or nitrates
Maintain cardiac output – vasopressors or inotropes; no consensus on best agent

## Slide 5
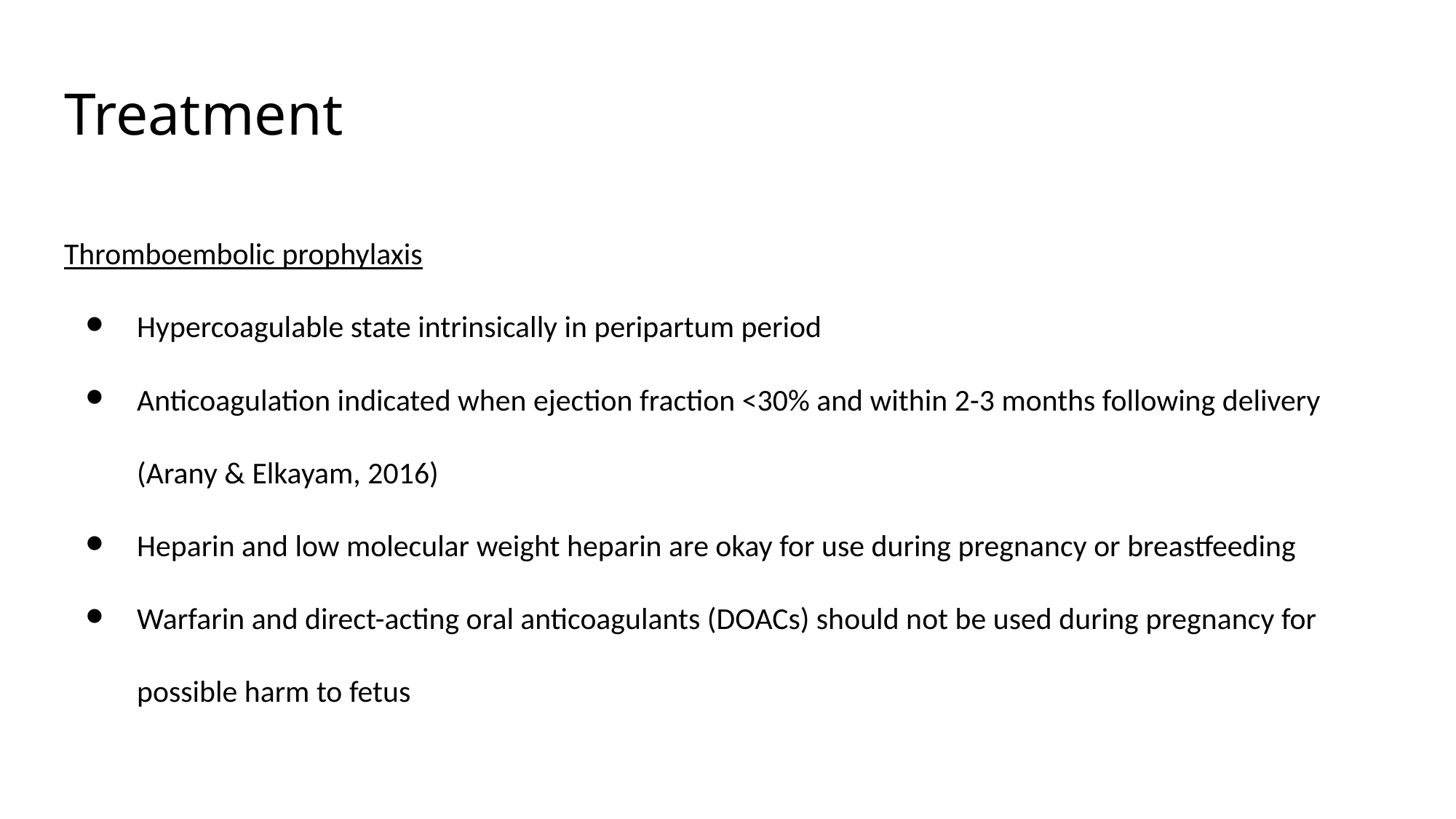

# Treatment
Thromboembolic prophylaxis
Hypercoagulable state intrinsically in peripartum period
Anticoagulation indicated when ejection fraction <30% and within 2-3 months following delivery (Arany & Elkayam, 2016)
Heparin and low molecular weight heparin are okay for use during pregnancy or breastfeeding
Warfarin and direct-acting oral anticoagulants (DOACs) should not be used during pregnancy for possible harm to fetus

## Slide 6
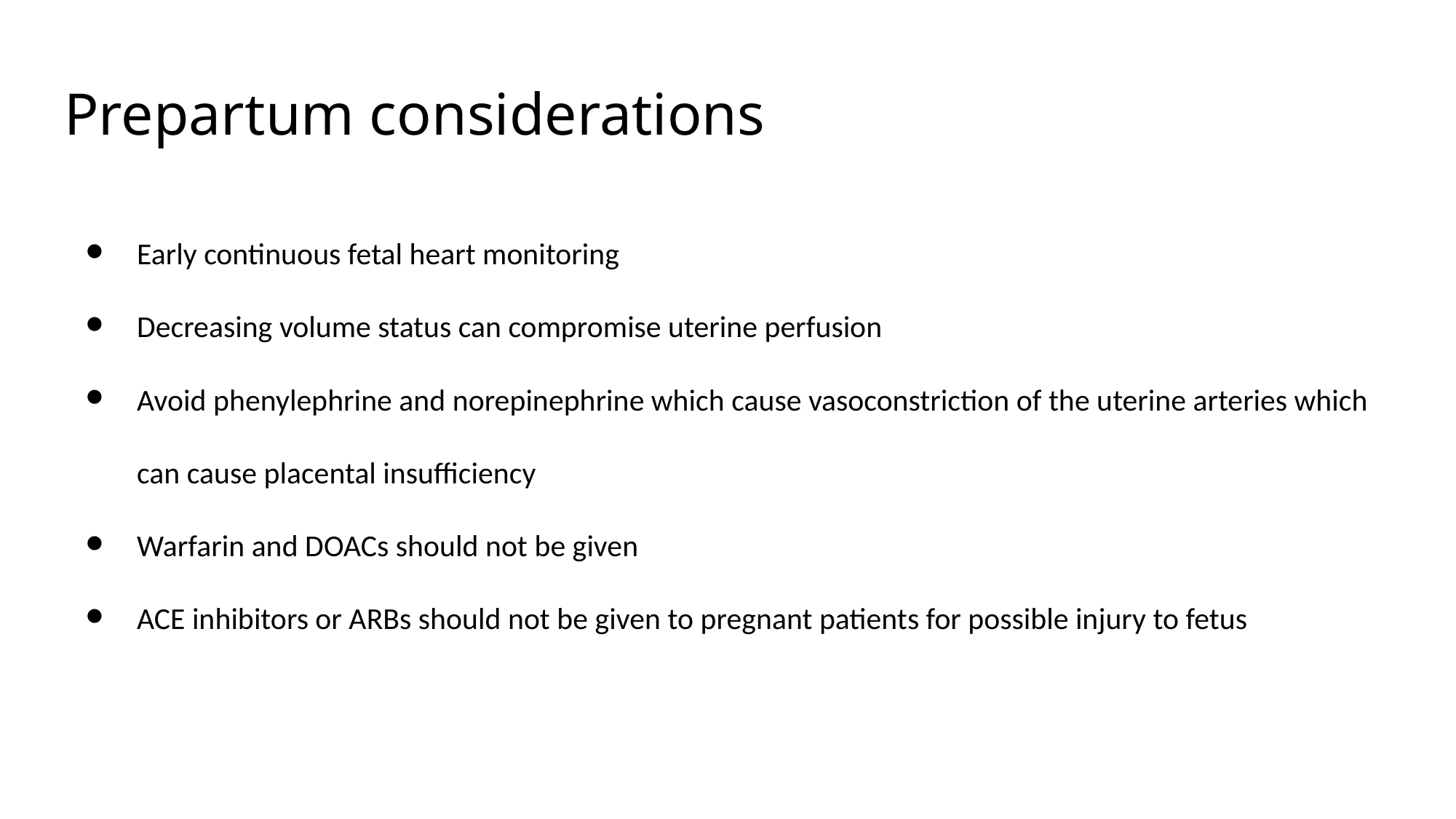

# Prepartum considerations
Early continuous fetal heart monitoring
Decreasing volume status can compromise uterine perfusion
Avoid phenylephrine and norepinephrine which cause vasoconstriction of the uterine arteries which can cause placental insufficiency
Warfarin and DOACs should not be given
ACE inhibitors or ARBs should not be given to pregnant patients for possible injury to fetus

## Slide 7
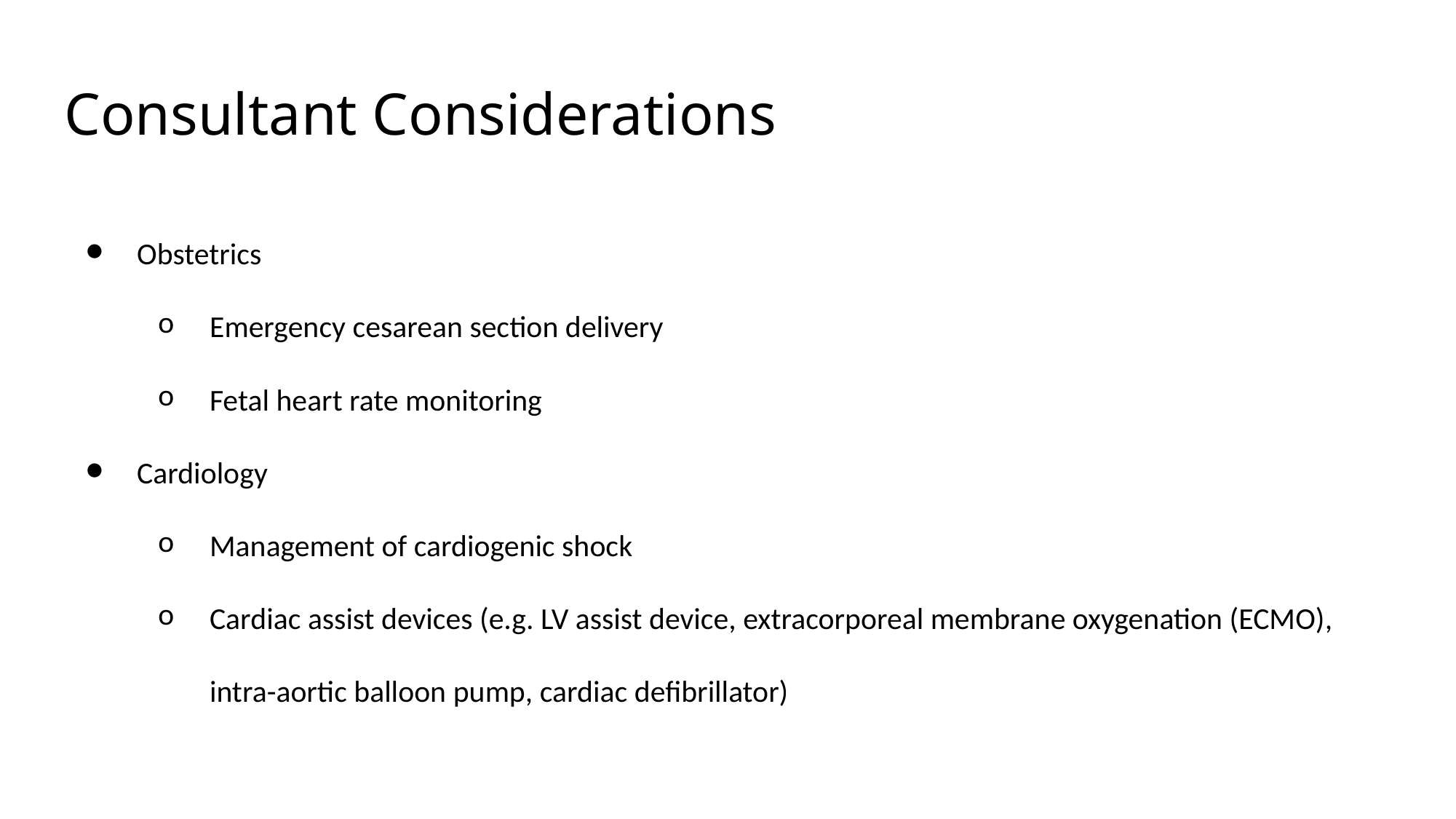

# Consultant Considerations
Obstetrics
Emergency cesarean section delivery
Fetal heart rate monitoring
Cardiology
Management of cardiogenic shock
Cardiac assist devices (e.g. LV assist device, extracorporeal membrane oxygenation (ECMO), intra-aortic balloon pump, cardiac defibrillator)
